# Polymer Simulations of Heteromorphic Chromatin Predict the 3D Folding of Complex Genomic Loci

**DOI:** 10.1016/j.molcel.2018.09.016

**Published:** 2018-11-15

**Authors:** Adam Buckle, Chris A. Brackley, Shelagh Boyle, Davide Marenduzzo, Nick Gilbert

**Affiliations:** 1 MRC Human Genetics Unit, MRC Institute of Genetics & Molecular Medicine, University of Edinburgh, Western General Hospital, Edinburgh EH4 2XU, UK; 2 SUPA, School of Physics and Astronomy, University of Edinburgh, Peter Guthrie Tait Road, Edinburgh EH9 3FD, UK

**Keywords:** chromatin, transcription, polymer modeling, histone modifications, genome organization, 3C, Capture-C, FISH, enhancer

## Abstract

Chromatin folded into 3D macromolecular structures is often analyzed by chromosome conformation capture (3C) and fluorescence in situ hybridization (FISH) techniques, but these frequently provide contradictory results. Chromatin can be modeled as a simple polymer composed of a connected chain of units. By embedding data for epigenetic marks (H3K27ac), chromatin accessibility (assay for transposase-accessible chromatin using sequencing [ATAC-seq]), and structural anchors (CCCTC-binding factor [CTCF]), we developed a highly predictive heteromorphic polymer (HiP-HoP) model, where the chromatin fiber varied along its length; combined with diffusing protein bridges and loop extrusion, this model predicted the 3D organization of genomic loci at a population and single-cell level. The model was validated at several gene loci, including the complex *Pax6* gene, and was able to determine locus conformations across cell types with varying levels of transcriptional activity and explain different mechanisms of enhancer use. Minimal *a priori* knowledge of epigenetic marks is sufficient to recapitulate complex genomic loci in 3D and enable predictions of chromatin folding paths.

## Highlights

• HiP-HoP: highly predictive heteromorphic polymer model to analyze chromatin structure
• Polymer simulations use widely available epigenetic and protein binding data as input
• Validate HiP-HoP model at complex loci using 3D FISH and Capture-C
• Simulations uncover striking conformational variability in chromatin fiber folding

## Graphical Abstract

**Figure:**
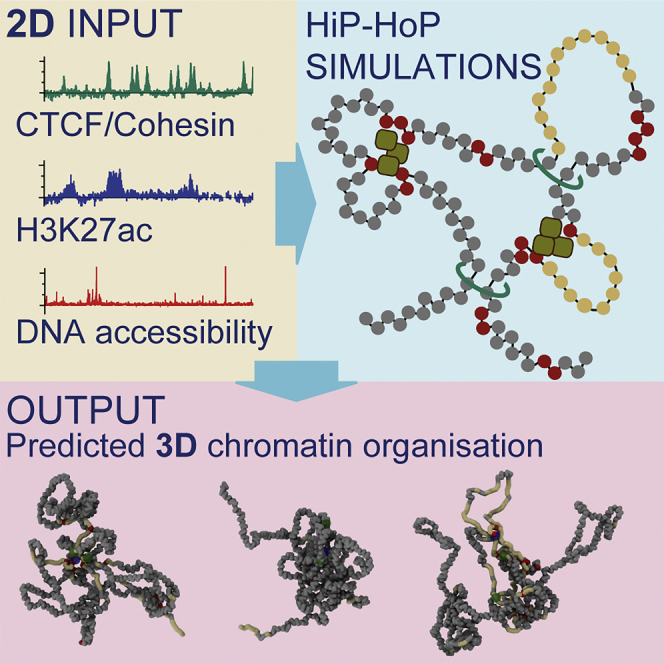

## Teaser

Buckle et al. develop a highly predictive heteromorphic polymer (HiP-HoP) model that uses epigenetic and protein binding data to predict 3D chromatin organizations, validated by FISH and Capture-C experiments. These simulations show striking conformation variability in fiber folding within a cell population and can explain different mechanisms of enhancer use.

## Introduction

Chromatin fiber folding in cells is dictated by a vast number of interactions between nucleosomes, chromatin-binding proteins, and structural components such as CCCTC-binding factor (CTCF)-cohesin loops, as well as the inherent structure of the underlying fiber. Chromatin is far from a bland homomorphic fiber; rather, it is a structurally heterogeneous material that is frequently disrupted at transcriptional hotspots (Gilbert et al., 2004, Naughton et al., 2010), and is thought to be locally compact in inactive regions. The ENCODE project (ENCODE Project Consortium, 2012) comprehensively mapped the distribution of epigenetic and structural features in different human and mouse cell lines. Many of these marks are surrogates for transcriptional activity that can impact local chromatin fiber structure. Previously, we developed a polymer modeling scheme based on the assumption that chromosome organization is driven by the formation of bridges by multivalent protein complexes (the transcription factor [TF] model; Brackley et al., 2013, Brackley et al., 2016a, Brackley et al., 2016b). For example, complexes of TFs and polymerase form enhancer-promoter interactions to organize active regions, while PRC (polycomb repressor complex) or HP1 proteins might arrange inactive and repressed regions. This model can predict large-scale organization, such as chromatin domains and compartments (Brackley et al., 2016b), and has been successful in describing some genomic loci at higher resolution (e.g., the α and β-globin loci; Brackley et al., 2016a). We also recently combined the TF model with the popular loop extrusion (LE) model for chromosome organization (Pereira et al., 2018), which explains features of chromatin loops mediated by cohesin and CTCF (Fudenberg et al., 2016, Sanborn et al., 2015). While this strategy successfully predicts large-scale features of genome organization, we show below that it cannot accurately predict the folding of the complex *Pax6* genomic locus at high resolution, which we probed experimentally at different levels using fluorescence *in situ* hybridization (FISH) imaging and Capture-C. *Pax6* is surrounded by constitutively expressed genes and multiple enhancers, providing a paradigm for complex genetic interactions (Buckle et al., 2018, Lacomme et al., 2018, McBride et al., 2011).

### Design

Our previous models use a simple bead-and-spring polymer to represent the chromatin fiber and as such assume that this has a uniform structure. We speculated that certain histone modifications would be indicative of disrupted chromatin with decreased linear fiber compaction; to this end, we developed a predictive heteromorphic polymer (HiP-HoP) model. Simulations of these “heteromorphic” chromatin fibers gave a much better recapitulation of locus conformation at transcriptionally active regions of the genome, providing a universal model for chromatin fiber folding that could potentially be applied to map 3D structures genome-wide in the future; as examples, here we studied the *Pax6*, globin, and *SOX2* loci. Unlike other widely used “inverse modeling” approaches for predicting 3D chromatin folding, such as the recent polymer-physics-based approach PRISMR (Bianco et al., 2018), or previous approaches based on Markov chain Monte Carlo or constrained molecular dynamics (Di Stefano et al., 2013, Di Stefano et al., 2016, Giorgetti et al., 2014, Zhan et al., 2017, Zhang and Wolynes, 2015), the present scheme does not rely on *any* fitting to preexisting chromosome conformation capture carbon copy (5C) or Hi-C data, making it applicable to a wider variety of experimental situations, for instance to investigate the 3D conformation of rare or hard-to-obtain tissues and cell types.

## Results

### Activity States of *Pax6* Show Differential Epigenetic Marks and CTCF Binding

In the present work, we set out to develop a universal approach for modeling chromatin fiber folding with limited experimental knowledge, based only on extensive freely available data generated from the ENCODE project. To develop this strategy, we investigated the folding of 5 Mb around the *Pax6* locus using three different immortalized cell lines that expressed *Pax6* at different levels (Figure S1), referred to as Pax6-OFF, ON, and HIGH cell lines. *Pax6* is flanked by two constitutively expressed housekeeping genes, with enhancer elements within the *Pax6* gene itself and at regions ∼50 kb upstream and ∼95 kb downstream; these are referred to below as the up- and downstream regulatory regions (URR and DRR, respectively) (Buckle et al., 2018, Kleinjan et al., 2006). The histone modification H3K27ac (Figure S1A), usually associated with enhancers and transcriptional activation, was found at the gene and distal enhancers when *Pax6* was active, and these regions at enhancers broadened significantly in HIGH-activity cells. Surprisingly, despite large differences in *Pax6* transcription, CTCF and Rad21 binding across the locus did not vary significantly between the cell lines (Figure S1A), but additional CTCF bound in close proximity to the *Pax6* promoters in *Pax6*-expressing cells.

### Active Epigenetic Marks Predict Locus Folding Only in Some Cell Lines

**Figure 1:**
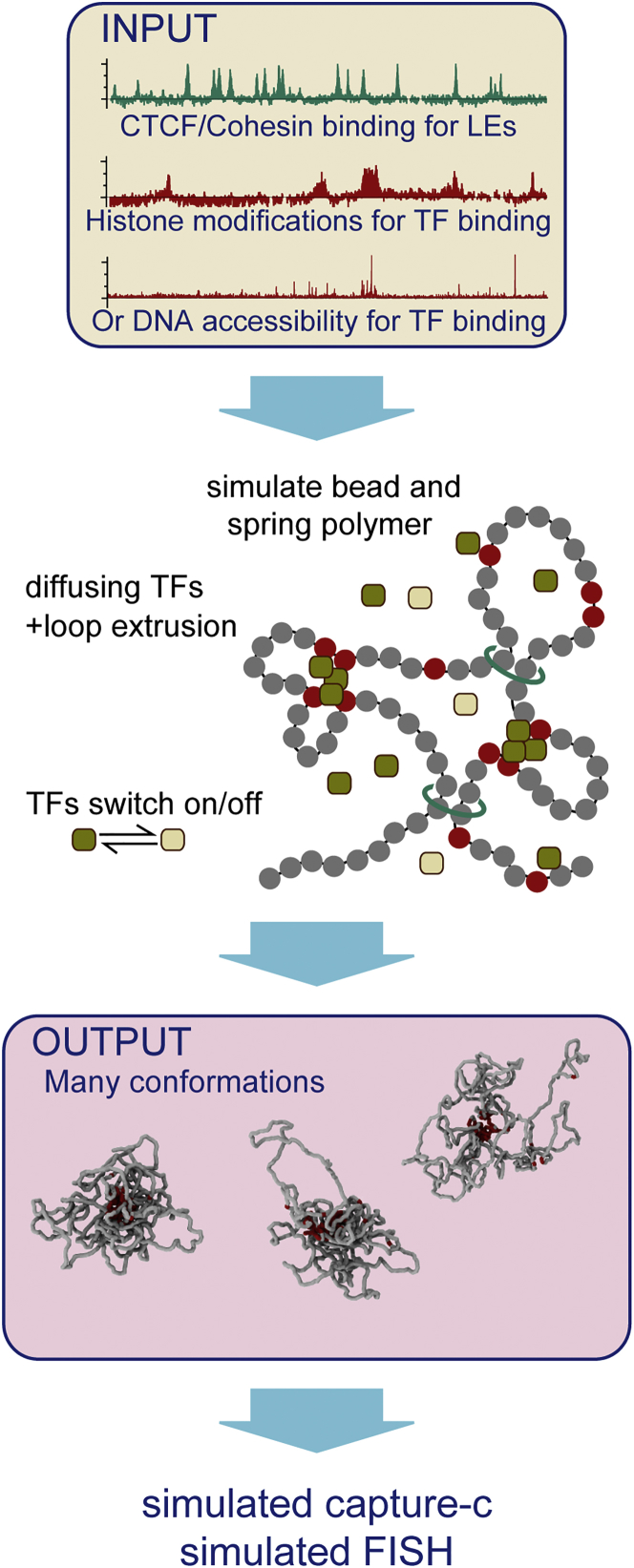
Bead and Spring Polymer for Modeling Chromatin Folding Schematic of the simulation model. A bead-and-spring polymer covered a 5-Mbp region around *Pax6*; each bead represented 1 kbp of chromatin. Initially, ChIP data for H3K27ac were used to color (mark) beads, and CCCTC-binding factor (CTCF)/Rad21 data were used to identify loop anchor beads. Later analysis used assay for transposase-accessible chromatin using sequencing (ATAC-seq) data to color beads. Freely diffusing beads represented TFs and bound colored polymer beads; these switched back and forth between a binding and non-binding state. LE factors (represented by additional springs in simulations, and shown as cyan rings) bind at adjacent polymer beads and extrude loops; extrusion was halted if the LE met an anchor bead that was orientated against the direction of extrusion. LEs were removed from (and returned to) the polymer stochastically at a constant rate. The model was used to generate a population of conformations from which simulated Capture-C and FISH measurements were obtained. Full details are given in STAR Methods. See also Figure S1.

Our previous modeling work (Brackley et al., 2016a, Brackley et al., 2016b, Pereira et al., 2018) gave good predictions of larger-scale (domain and compartment level) chromosome organization. To test whether this scheme can also predict folding of complex genetic loci at higher resolution, we performed simulations for the *Pax6* locus. This model, where a chromosome region is represented as a bead-and-spring polymer (with each bead representing 1 kb), combines two views on what drives chromatin conformation (see Figure 1 for a schematic). First, the TF model (Brackley et al., 2016a, Brackley et al., 2016b) postulates that promoter-enhancer interactions are mediated by diffusing protein complexes which form molecular bridges between their binding sites. Here, we began by assuming that TFs bind H3K27ac regions and switch back and forth between a binding and a non-binding state. Switching models post-translational modifications, active protein degradation, or programmed polymerase unbinding after transcription termination (Brackley et al., 2017, Pereira et al., 2018); it enables simultaneously strong TF binding and fast turnover of bound TFs (as observed by photobleaching experiments; Liu and Tjian, 2018), and drives the system away from equilibrium. Second, the LE model (Fudenberg et al., 2016, Sanborn et al., 2015) views cohesin and CTCF as the structural organizers of the genome, with cohesin forming chromatin loops via an extrusion mechanism that could be transcription dependent (Racko et al., 2018). LEs stop if they encounter a CTCF site that has a binding motif oriented toward the direction of extrusion; this enables stable looping between CTCF sites with binding motifs that are in a convergent, but not divergent, arrangement (Rao et al., 2014). We used the model to generate an ensemble of locus conformations representing a population of cells and from this extracted both chromosome conformation capture (3C)-like information and single-cell simulated FISH data (see STAR Methods for details of the combined TF + LE simulation scheme).

**Figure 2:**
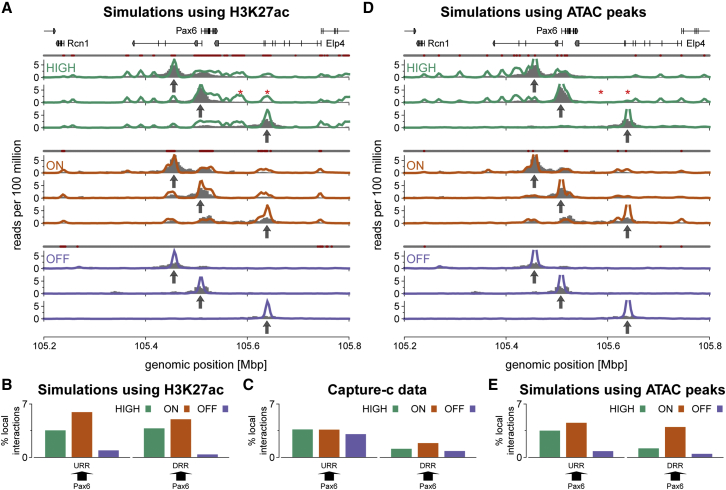
Polymer Simulations Predict Chromosome Interactions in Different Cell Lines (A) Simulated Capture-C profiles (colored lines) are shown for three cell lines, for three different viewpoints (at the URR, DRR, and *Pax6*, indicated by arrows). The corresponding experimental data are shown as gray bars. Above each set of plots, a line of points show how beads were colored as non-binding (gray) or binding (red) for TFs. In simulations, *Pax6* interacts strongly with a broad acetylated region downstream of the gene (red stars); this is not observed in the experimental data. (B) Plot showing the level of interaction between a viewpoint and a specified 10-kbp region. The arrow indicates the viewpoint (arrow base), and the interacting region (arrowhead). The height of the bar shows the number of interactions with the 10-kbp region as a percentage of interactions with the locus as a whole (chromosome 2 [chr2]: 105,200,000–105,800,00). (C) Similar plots were obtained from experimental Capture-C data. (D) Simulated Capture-C profiles for a model where ATAC-seq data (Figure S1D) were used to infer TF-binding sites instead of H3K27ac. The erroneous interactions marked in (A) are now absent (red stars). (E) Similar plots to those in (B) but from the simulations using ATAC-seq data. See also Figure S2.

To validate the model, we used the Capture-C protocol (a combination of 3C and oligonucleotide capture followed by high-throughput sequencing; Hughes et al., 2014) to obtain interaction profiles from a set of probes, or “viewpoints” across the locus (see STAR Methods and Figure S2). The simulations gave good predictions of chromatin interactions in the OFF and ON cell lines (Figure 2A); they showed that in ON cells, the *Pax6* promoters interact with both distal enhancers. However, notably, the model failed to correctly predict chromatin contacts in the HIGH-activity cells (Figures 2B and 2C).

### DNA Accessibility Better Predicts Locus Folding

In Pax6-HIGH cells, there was a reduction in looping to the DRR compared to ON cells, despite a broadening of the H3K27ac mark; this is inconsistent with the typical looping model for enhancer action and is not correctly predicted by simulations (which did show promoter-enhancer loops, as well as interactions with other acetylated regions between *Pax6* and the DRR; Figure 2A, red stars). From this analysis, a simple promoter-enhancer looping mechanism for regulating chromatin folding does not occur. Our previous studies on globin loci (Brackley et al., 2016a) use DNA accessibility data as a proxy for protein binding. In addition to mapping epigenetic marks, ENCODE has extensively characterized chromatin disruptions using both DNaseI sensitivity and assay for transposase-accessible chromatin using sequencing (ATAC-seq); to determine if this information gave improved model predictions, we generated ATAC-seq data on the three cell lines (Figure S1D). This revealed that in ON cells, there is an ATAC peak within the DRR, whereas in Pax6-HIGH cells, this peak is absent (despite the broadening of the H3K27ac mark). Simulations predicated on TFs only binding to ATAC peaks gave better predictions of Capture-C data in all three cell lines (Figures 2D and 2E).

These results show that while histone modification data can be used to recover the large-scale domain structure of chromosome organization (Brackley et al., 2016b, Pereira et al., 2018), for more complex loci at higher resolution, DNA accessibility gives a better prediction of TF-binding-driven structure. The molecular basis for this is unclear but probably reflects a more direct correlation between TF binding and disrupted chromatin, while histone modifications are generated indirectly as a consequence of acetyltransferases or methylases being recruited to chromatin.

### High-Level Transcription Drives Local Decompaction

**Figure 3:**
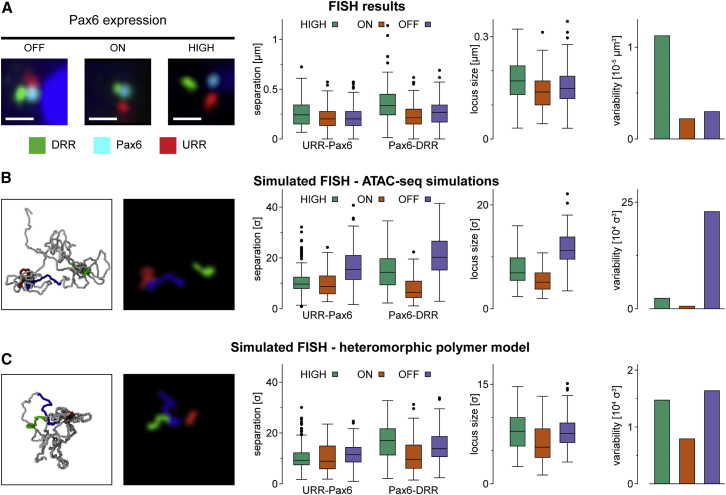
Fluorescence Microscopy Gives Single-Cell Information on Locus Conformation (A) 3D FISH experiment using probes positioned at the URR, DRR, and *Pax6* gene (Figure S3A). Left: representative three-color 3D FISH images. Scale bar, 0.5 μm. Mid-left: distributions of probe separations shown as boxplots. Mid-right: boxplots showing the size of the locus calculated from three-color FISH experiments. Right: bar graph displaying variability in locus conformation (see STAR Methods and Figure S3E). In general, the locus becomes more compact in Pax6-ON cells compared to Pax6-OFF cells but becomes less compact (and more variable) in Pax6-HIGH cells. (B) Simulated FISH data extracted from locus conformations generated by chromatin folding simulations using ATAC data to define TF-binding sites (as shown in Figures 2D and 2E). Left: representative snapshot of the locus conformation alongside a simulated FISH image shown for illustrative purposes. Mid-left: boxplots showing distances between probes given in simulation units (σ) (see STAR Methods). Right: locus size and structural variability as in (A), but data are given in simulation units. These simulations depart from the experimental measurements, as the Pax6-OFF cells are the most decompacted and highly variable. (C) Simulated FISH measurements from the heteromorphic polymer model. Agreement with the experimental data improves in that the locus is decompacted in Pax6-HIGH cells and the variability is far larger in the highly expressing cells. See also Figure S3.

From the panoply of simulated structures, individual measurements can be extracted equivalent to information obtained from FISH experiments (Figure 3). This provides values for the distance between pairs of points on the polymer but also enables the volume of the locus to be predicted. To validate these results, we performed 3D FISH experiments (Figures 3A and S3A–S3C). Initially, compaction upstream and downstream of the *Pax6* locus was measured (using pairs of FISH probes at URR/Pax6 and Pax6/DRR, respectively). Both probe pairs showed a non-monotonic variation in separation as a function of *Pax6* activity, both *in vivo* and in simulations. Separations were reduced in Pax6-ON compared to Pax6-OFF cells but were larger in Pax6-HIGH cells than in Pax6-ON cells. To quantify how well the simulations predicted the data, a measure called the K-score, ranging from zero to one (from no agreement to perfect overlap of separation distributions), was defined (see STAR Methods and Figure S3D); despite a high score of K = 0.70, simulations did not correctly predict the result that Pax6-HIGH cells showed significantly larger probe separations than Pax6-OFF cells. Varying the bead size in the simulations to either 400 bp or 3 kb further reduced the K-score (Figure S3D).

To test how the simulations predicted overall locus volume, the volume enclosed by three FISH probes was experimentally measured (URR/Pax6/DRR). Our previous studies (Naughton et al., 2010) showed that transcriptionally active regions are decompacted; surprisingly, the simulations predicted that the HIGH cells would be more compact than Pax6-OFF cells (Figure 3B). This was not consistent with values obtained from 3-probe FISH (Figure 3A; note also that again a non-monotonic trend through OFF-ON-HIGH cells was observed). We also designed a measure of cell-to-cell variability by computing the level of the scatter in a plot where simultaneous URR-Pax6, Pax-DRR, and URR-DRR measurements were shown on three axes (Figures S3E and S3F; see STAR Methods). Simulations predicted that Pax6-OFF cells would show most variability; again, this was inconsistent with 3-probe FISH, where Pax6-HIGH cells showed the most variability.

### Polymer Simulations of Heteromorphic Chromatin Fibers Predict Experimental Data

**Figure 4:**
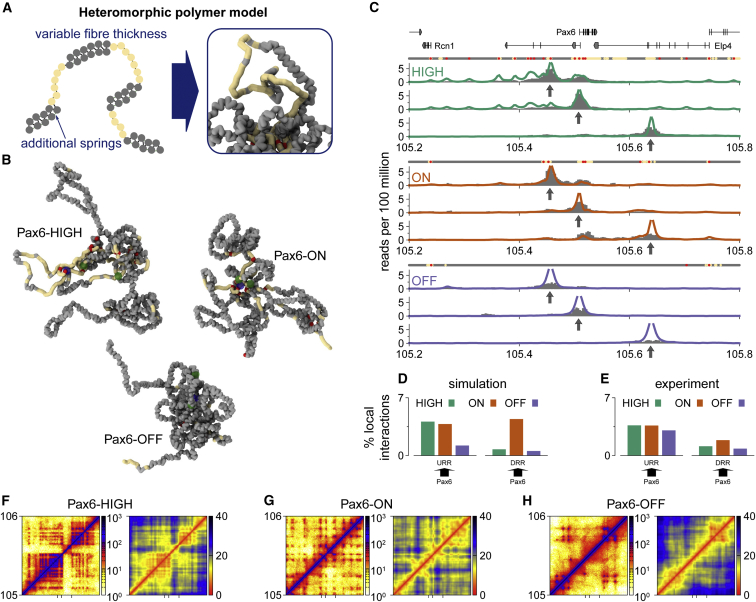
Heteromorphic Chromatin Fiber Model Gave Better Predictions of Experimental Observations For a Figure360 author presentation of Figure 4, see https://doi.org/10.1016/j.molcel.2018.09.016. (A) Left: schematic showing how two levels of chromatin fiber thickness were simulated by adding additional springs between next-nearest neighboring beads. Regions that have the H3K27ac mark (yellow) did not have these extra springs. Diffusing TFs and LEs were then added as before. Right: snapshot of a typical simulated fiber; red regions correspond to TF-binding sites as inferred from ATAC peaks. TFs are not shown for clarity. (B) Typical snapshots of the simulated fiber in each of the three cell types. Only the *Pax6* locus (chr2, 105–106 Mb) is shown; TFs are not shown. Yellow and red regions indicate H3K27ac and ATAC regions, respectively. The transparent blue sphere indicates the *Pax6* promoter, and green spheres indicate the URR and DRR. (C) Simulated Capture-C tracks from the three cell types (solid lines) for three viewpoints (positions indicated with arrows); gray profiles show experimental data. Viewpoints (URR, *Pax6*, and DRR) are indicated with arrows, and the bead colors are indicated by rows of points above each set of plots; gray regions have a more compact fiber, yellow indicates a more open H3K27ac-marked fiber, and red indicates ATAC-seq peaks (TF binding). (D and E) Bar plots as in Figure 2B showing simulated (D) and experimental (E) interactions between specified viewpoints (arrow base) and a 10-kbp region around a feature of interest (arrowhead). The trends seen in the experimental data (E) are now better predicted by the simulations. (F–H) Left: simulated Hi-C maps for Pax6-HIGH (F), Pax6-ON (G), and Pax6-OFF (H) cells. Right: a similar map shows the mean distance between each bead in the region in simulation length units (σ). Ticks on the horizontal axis indicate the positions of the URR, *Pax6* promoters, and DRR. See also Figures S4 and S5 and Videos S1 and S2. Figure360: An Author Presentation of Figure 4

The model described above agreed with population based 3C-style data but could not accurately predict trends observed in single-cell FISH. We reasoned that because our model assumed a homomorphic fiber, variation between cell types could only arise from differences in the TF binding or CTCF locations as loop extruder anchor sites. However, it is known that chromatin fibers can adopt alternate configurations (Florescu et al., 2016, Gilbert et al., 2004, Gilbert and Allan, 2001, Naughton et al., 2010), and recent radiation-induced spatially correlated cleavage of DNA with sequencing (RICC-seq) experiments suggest there are two main local structural motifs associated with chromatin fibers: a more open and a more compact conformation (Risca et al., 2017). It has also been suggested that acetylation marks regions of disrupted chromatin (Hebbes et al., 1988, Hebbes et al., 1994, Risca et al., 2017), and indeed RICC-seq data showed a more open chromatin structure was correlated with H3K27ac. Consistent with this, volumes measured for individual FISH probes were correlated with the level of H3K27ac within the probe region (Figures S3G and S3H). Therefore, we hypothesized that including a different chromatin fiber structure at H3K27ac regions might improve the agreement between simulations and FISH data. To achieve this in a simple way, additional springs were introduced to regions that do not have the acetylation mark (i.e., where the fiber had a higher linear compaction [kbp of DNA in a given length of fiber]), leaving H3K27ac regions less compact (see schematic in Figure 4A and STAR Methods for details). We call this the highly predictive heteromorphic polymer (HiP-HoP) model.

Since we do not know how chromatin structure actually varies along the fiber at high resolution, and in reality there is likely to be more than two levels, we could not expect the model to predict the FISH data exactly. Nevertheless HiP-HoP simulations did correctly reproduce all observed trends in our experiments (Figures 3C and S3I–S3K). Most notably, we found that with this model, the Pax6/DRR separations were on average furthest apart in the Pax6-HIGH cells, and this cell type also showed much higher cell-to-cell variability than in the previous simulations. The extent of chromatin decompaction in Pax6-HIGH cells is apparent from inspection of simulation snapshots of the locus structure (Figure 4B; Videos S1 and S2). The K-score (Figure S3D) increased to 0.77 (in comparison to a score of 0.70 for the simulations shown in Figure 3B or 0.58 for a randomized control; see STAR Methods), indicating improved agreement with the experimental data (Figure 3A and Figures S3I–S3K). We defined a second quantitative metric, the Q-score, which measures the agreement between experimental and simulated Capture-C profiles (Figure S4A); loosely this can be interpreted as the proportion of Capture-C peaks that are correctly predicted by the model. Although we noted very little visible change in the simulated Capture-C profiles, the Q-score increased from 0.45 (for the simulations shown in Figure 2D) to 0.51 (Figure 4C); this is compared to a value of 0.35 obtained in a randomized control (see STAR Methods and Figures S4A and S4B), and there was qualitative agreement between URR and DRR interactions with *Pax6* (Figures 4D–4E).

Video S1. Typical Simulated Locus Structures Are Shown for Each of the Three Cell Types, Related to Figure 4 In each case, the region chr2:105,000,000-106,000,000 is shown. Beads that overlap peaks of H3K27ac are shown in yellow; beads at the Pax6 promoters, the URR, and the DRR are shown in red, blue, and green, respectively. All other beads are gray.

Video S2. A Portion of the Simulation Dynamics Is Shown for a Single Simulation from Each Cell Type, Related to Figure 4 As in Video 1, only the region chr2:105,000,000-106,000,000 is shown, beads that overlap peaks of H3K27ac are shown in yellow, and beads at the Pax6 promoters, the URR, and the DRR are shown in red, blue, and green, respectively.

Although the Capture-C protocol only gives information about interactions for specific probed “viewpoints,” a signature of a domain structure was present in the data as we observed that probes from the left of the locus interact more with regions toward the right, and vice versa (Figure S2E). The same directional bias was found in simulated Capture-C data for Pax6-HIGH cells but was not present in the other two cell lines (Figure S4C). However, a full Hi-C-like interaction map can be extracted from the simulations, and these indeed showed domains for all cell types (Figures 4F–4H and S4D). A reason for the seemingly different signals might be that the capture targets (viewpoints) tend to be positioned on TF-binding beads, and overestimation by the HiP-HoP model of long-range interactions at these sites may skew the directionality metric.

The simulation scheme also allows investigation of different scenarios for genome organization. For example, simulations can be performed with different aspects of the model switched off (Figures S5A–S5E). We found that switching off LEs (similar to a knockdown of cohesin or its loader, leaving only diffusing TFs binding to ATAC peaks and the heteromorphic polymer based on H3K27ac data) led to a reduction in agreement with data (the Q-score was reduced by 20% [Figure S4A] and the FISH K-score by 9% [Figure S3D]). At a larger scale, the simulated Hi-C maps changed; they became more “spotty” (showing promoter-enhancer interactions) and the domains less prominent (Figure S5B). If instead TFs are removed from the model (keeping LEs and the heteromorphic polymer), the domains remain, but most enhancer-promoter interactions disappear (Figure S5C); also, the Q-score is reduced by ∼15% compared to the simulations in Figure 4, although the K-score shows a small increase of ∼2%. This points to a scenario where TFs give rise to promoter-enhancer interactions, while LEs generate domains.

### HiP-HoP Simulations Reveal Multiple 3D Chromatin Structures for the *Pax6* Locus

**Figure 5:**
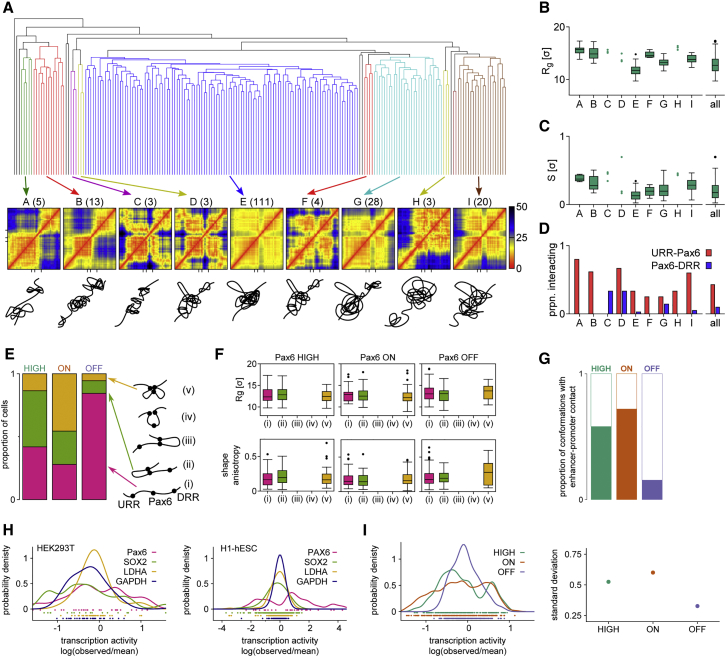
Hierarchical Clustering Analysis of 200 Simulated Pax6-HIGH Conformations Revealed Groups of Similar Structures (A) Top: dendrogram generated via hierarchical clustering using an “average” linking criterion. A Euclidean distance metric based on the pairwise difference between the separations of all pairs of beads was used (see STAR Methods). Some groups of similar conformations are highlighted in color. Middle: for each highlighted group, a distance map of the region chr2:105,000,000–106,000,000 is shown (color scale gives the distance between pairs of beads in simulation distance units (σ)). Axis ticks on the bottom of the plots show the positions of the URR, *Pax6* promoters, and the DRR. Bottom: sketched representations of potential combinations. (B) The distribution of the radius of gyration of the region chr2:105,000,000–106,000,000 is shown as a boxplot for groups highlighted in (A). This is a measure of the size of the locus. Where there are fewer than four conformations in a group, single points are shown. To the left, the distribution for all 200 conformations is shown. (C) Similar plot to (B) but showing the shape anisotropy, a measure of the relative shape of the locus. This ranges from 0 for a spherically symmetric arrangement to 1 for a linear arrangement. (D) For each group of conformations highlighted in (A), the proportion in which *Pax6* interacts with each of the distal regulatory regions is indicated. As expected from the simulated Capture-C data shown in Figure 3, in Pax6-HIGH cells, interactions with the DRR are rare. (E) Bar plot showing the proportion of conformations falling into each of the following groups: (1) *Pax6* does not interact with either distal regulatory region; (2) *Pax6* interacts with the URR; (3) *Pax6* interacts with the DRR; (4) the URR interacts with the DRR, but not with *Pax6*; (5) all three regions interact simultaneously. Schematics are shown to the left. Note that there are no conformations in groups 3 or 4 in any of the cell types. (F) Radius of gyration and shape anisotropy measurements for the groups in (E). Anisotropy is a measure of how sphere-like (values close to zero) or rod-like (values close to 1) the region is. (G) Graph showing the proportion of conformations showing an interaction between the *Pax6* promoter and one or more enhancers were determined for each cell line. (H) Single-cell transcriptional activity for *PAX6*, *SOX2*, *LDHA*, and *GAPDH* in HEK293T (left, GEO: GSE67835; Darmanis et al., 2015) and H1 human embryonic stem cells (H1-hESCs) (right, GEO: GSE64016; Leng et al., 2015). Individual data points are shown below the graphs. (I) Left: simulated transcriptional variability of Pax6-HIGH, ON, and OFF cells. Data points for individual structures (A) are drawn below the graph. Right: SD (heterogeneity) of distributions shown in the left panel. See also Videos S3, S4, and S5.

Experimental and simulated FISH measurements suggest that there is substantial variation in the distribution of interprobe distances in Pax6-HIGH cells. More detailed information on structural variability is best extracted from an analysis of individual simulated structures, as these give information about chromatin fiber conformation at high resolution (single monomer, or 1 kbp). We focus here on the case of Pax6-HIGH cells. First, a qualitative inspection of simulated conformations (see Videos S3, S4, and S5) provided a striking visual impression of the large structural diversity in *Pax6* folding; it was apparent that the shape and size of the locus varies widely. Second, a clustering analysis of all simulated structures based on mean-squared differences of monomer separations showed that there are multiple typical structures for the chromatin fiber around *Pax6*, with several possible structure classes (Figure 5A). The distribution of locus size and shape (quantified via the radius of gyration and shape anisotropy) showed only slight variation between each class (Figures 5B and 5C); the largest difference is between class A, where the locus is usually larger and more elongated, and class E, where the locus is smaller and more spherical. Intriguingly, each class contained some structures where *Pax6* and its distal enhancers are in contact and some where they are far apart (Figure 5D). These two motifs are likely to be associated with different levels of transcriptional activity, yet our analysis shows that this is largely independent of the larger-scale structure of the locus. While a similar clustering analysis has pointed to structural diversity in the folding of other chromatin loci, the extent of variability found in the *Pax6* locus exceeds that observed in less complex loci such as globin (where simulations suggested the existence of two main classes of structures; Brackley et al., 2016a).

Video S3. Snapshots of 200 Configurations of the Locus (region chr2:105,000,000–106,000,000) for Pax6-HIGH Cells Are Shown, Related to Figure 5 Beads that overlap peaks of H3K27ac are shown in yellow; beads at the Pax6 promoters, the URR, and the DRR are shown in red, blue, and green, respectively. All other beads are gray. In the top left, the same snapshot is shown, but now all beads are invisible except those at the URR, Pax6, and the DRR. The 3D structure has been orientated such that the triangle made by the URR-Pax6-DRR beads is in the plane of the image. The order in which the structures are shown is such that the conformation that most closely matches the current one is shown next (using the same distance metric as in the hierarchical clustering analysis).

Video S4. Snapshots of 200 Configurations of the Locus (Region Chr2:105,000,000–106,000,000) for Pax6-ON Cells, Related to Figure 5 Beads that overlap peaks of H3K27ac are shown in yellow; beads at the Pax6 promoters, the URR, and the DRR are shown in red, blue, and green, respectively. All other beads are gray. In the top left, the same snapshot is shown, but now all beads are invisible except those at the URR, Pax6, and the DRR. The 3D structure has been orientated such that the triangle made by the URR-Pax6-DRR beads is in the plane of the image. The order in which the structures are shown is such that the conformation that most closely matches the current one is shown next (using the same distance metric as in the hierarchical clustering analysis).

Video S5. Snapshots of 200 Configurations of the Locus (Region Chr2:105,000,000–106,000,000) for Pax6-OFF Cells, Related to Figure 5 Beads that overlap peaks of H3K27ac are shown in yellow; beads at the Pax6 promoters, the URR, and the DRR are shown in red, blue, and green, respectively. All other beads are gray. In the top left, the same snapshot is shown, but now all beads are invisible except those at the URR, Pax6, and the DRR. The 3D structure has been orientated such that the triangle made by the URR-Pax6-DRR beads is in the plane of the image. The order in which the structures are shown is such that the conformation that most closely matches the current one is shown next (using the same distance metric as in the hierarchical clustering analysis).

Another way to group the simulated structures is to directly consider the interactions between the distal regulatory regions and the *Pax6* promoters (Figure 5E). There were five possible combinations: none of the elements were in contact, *Pax6* contacted one of the enhancer regions, the two enhancer regions were in contact with each other (but not *Pax6*), or all three were in contact. Interestingly, within the 200 structures for each cell type, none had interactions between the two enhancers without them also interacting with *Pax6*, and the DRR never interacted with *Pax6* in isolation. Consistent with the observations detailed above, in Pax6-OFF cells, the majority of conformations had no Pax6-enhancer interactions; in Pax6-ON cells, a large proportion of conformations had *Pax6* interacting with both enhancers; and in Pax6-HIGH cells, *Pax6* more often only interacted with the URR (Figure 5E). Consistent with the clustering analysis, the *Pax6* interactions did not depend on the size or shape of the locus as a whole (Figure 5F).

Recent studies have shown that transcription is dependent on promoter-enhancer interactions (Brackley et al., 2016a, Chen et al., 2018, Gu et al., 2018); consequently, Pax6-HIGH and Pax6-ON cells had far more promoter-enhancer interactions than Pax6-OFF cells (Figure 5G). As single-cell transcription data indicate that there is more transcriptional heterogeneity in genes expressed from complex loci such as *PAX6* and *SOX2* compared to housekeeping genes (e.g., *GAPDH* and *LDHA*) (Figure 5H), we speculated that the number of transcriptional states (Figure 5A) and the interaction between promoters and enhancers (Figure 5E) from our simulations might reflect transcriptional heterogeneity. To assess this, we defined a transcriptional activity score for each simulated conformation that is based on proximity of promoters and enhancers (see STAR Methods). Pax6-ON and Pax6-HIGH cells were highly transcriptionally heterogenous (Figure 5I), analogous to the single-cell data for complex versus housekeeping loci, suggesting that structural simulations might give some insight into potential transcriptional heterogeneity.

### Application of HiP-HoP Simulations to Active Chromatin Loci

Above, we focused on *Pax6* folding, but since our predictive heteromorphic polymer simulation approach only requires four different datasets as input (DNA accessibility, histone acetylation, and CTCF/Rad21), it is applicable to a large number of active chromatin loci in different cell lines (associated with different levels of locus activity). To test how well the HiP-HoP model performs at other loci, we studied the folding of the α and β-globin loci in mouse erythroid cells, which involve simpler genomic interactions with respect to *Pax6*. As expected, our simulated structures for globin compared favorably with previously published Capture-C and FISH data (Figure S6).

**Figure 6:**
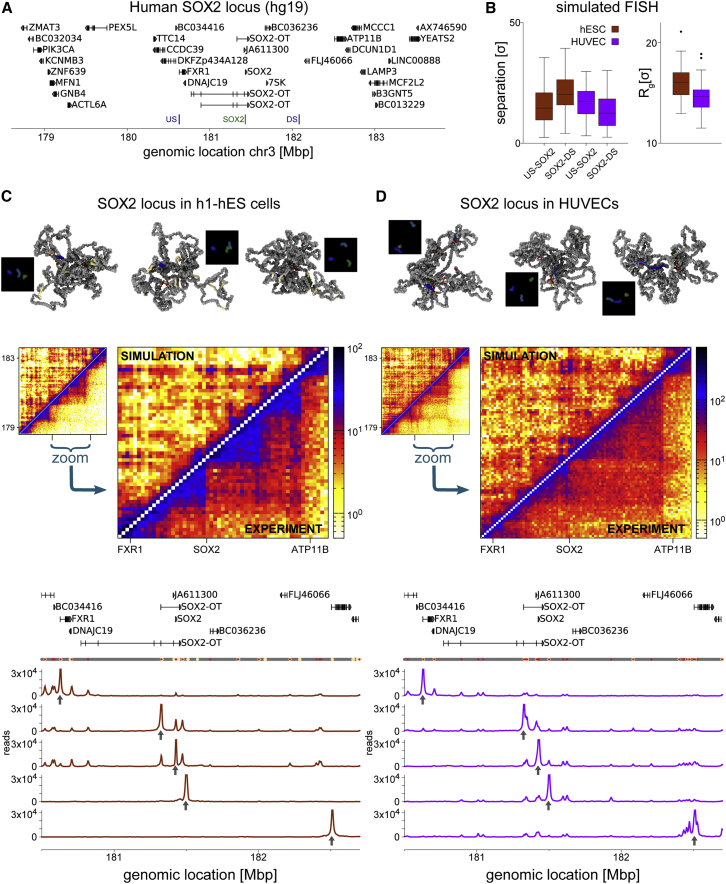
Chromatin Simulations of a 5-Mbp Region around the Human *SOX2* Locus in H1-hESCs and HUVECs (A) A map of the locus is shown, with the positions of three “simulated” FISH probes indicated. (B) Boxplots showing simulated FISH results, and the radius of gyration of region for each cell type. (C) Top: example conformations from hESC simulations alongside simulated microscopy images. Middle: Hi-C map of the simulated region, and a zoom around the *SOX2* gene. The upper triangles show Hi-C data from (Dixon et al., 2012), and the lower triangles shows simulated maps. Bottom: simulated Capture-C data from four simulated viewpoints (indicated by arrows). (D) Similar plots to (C) but from HUVEC simulations. The Hi-C data are from Rao et al. (2014). See also Figure S6.

To show that the HiP-HoP model can work in different organisms, we also considered the human *SOX2* locus, a key reprogramming gene (Figure 6). We found good agreement with experimental Hi-C contact maps in stem cells and umbilical vein epithelial cells (HUVECs). These results show that our model is portable to other loci and that it can be used to predict folding of loci that have not yet been investigated either by chromosome conformation capture or FISH.

## Discussion

In this study, we developed a simulation model of 3-D genome organization treating chromatin as a heteromorphic polymer, where the H3K27ac histone modification is associated with a locally disrupted chromatin fiber; this was corroborated by a strong positive correlation between histone acetylation and putative regions of disrupted chromatin based on RICC-seq experiments (Hebbes et al., 1994, Risca et al., 2017). The failure of simple models and the development of complex simulations led to greater understanding of the *Pax6* locus; a substantial increase in *Pax6* expression observed in the HIGH-activity cells is *not* accompanied by an increase in looping interactions between the *Pax6* promoters and the DRR (a change that is observed when going from Pax6-OFF to Pax6-ON cells). Additionally, in Pax6-HIGH cells, microscopy and HiP-HoP simulations showed that the chromatin fiber at the DRR must undergo a dramatic decondensation, which is associated with a 50% increase in the mean separation between this enhancer site and the *Pax6* promoter. Two possible explanations for these results are that the downstream enhancer is not involved in upregulation of *Pax6* in these cells or that there is an enhancer activity that does not require physical proximity to the promoter but is instead associated with chromatin decompaction (Benabdallah et al., 2017). One can speculate on possible mechanisms through which decompaction of an enhancer region might lead to upregulation of a nearby gene. Perhaps the region adopts a fiber structure that can more readily accommodate supercoiling generated by a transcribing polymerase; alternatively, transcription at the enhancer itself might lead to a localization of activating proteins that facilitates transcription at the promoter; or perhaps the expansion of the enhancer region alters the dynamical properties of the wider locus. Whatever the mechanism, an interesting feature at *Pax6* is that the DRR seems to operate differently in different cell lines, which is an area for future research.

These simulations also suggested that for *Pax6* (and possibly other complex loci), there is a large degree of cell-to-cell variation in locus conformation, and this probably reflects how cells require different transcription levels and heterogeneity in various cellular situations, regulated by alternate enhancers. This is different to the case of the globin loci where both loci were found to adopt one of a small number of preferred configurations (Brackley et al., 2016a). Earlier computational models failed to predict trends in the FISH data but gave similar predictions for Capture-C profiles; this highlights a potential issue for approaches that generate conformations based on fitting to existing Hi-C data, where more than one solution may be possible.

### Limitations

There are currently a number of limitations to the HiP-HoP framework that could be further developed in the future. First, it is possible to include proteins and binding sites corresponding to inactive marks, such as H3K27me3 or H3K9me3, which are associated with facultative and constitutive heterochromatin, respectively. While these marks are relatively rare in active loci such as those we have considered here, we expect they will be needed to get a complete picture of locus folding and a full agreement with experiments. Second, it would be of interest to ask whether including more levels of local chromatin compaction affects the level of quantitative agreement with experiments, selected for instance by analyzing a set of histone modifications rather than the single acetylation mark we considered here. On a similar note, as previously done for simpler models (Brackley et al., 2016a), it is in principle possible to combine the ATAC-seq data with chromatin immunoprecipitation sequencing (ChIP-seq) or bioinformatic analyses of specific TF binding to determine more precisely the pattern of binding sites on chromatin, if more information on the identity of the TFs regulating the genes within a locus is known.

Additionally, while HiP-HoP was used in this work to characterize static 3D chromatin structure, either at the population or single-cell level, it is also possible to extract dynamical information and predict chromatin mobility in different active loci, thereby providing a more direct link between structure and transcription which could be tested by future high-resolution microscopy of chromatin dynamics in live cells.

### Conclusions

In the future, it would be informative to apply HiP-HoP to many different loci in different cell types to understand the organizational principles of different classes of gene. We also expect the model to be readily extended to account for colocalization of repressed regions. As input to HiP-HoP is based on widely available datasets (ATAC or DNAase-seq for TF binding, H3K27ac to predict disrupted chromatin regions, and ChIP data for CTCF and the cohesin subunit Rad21 to define loop anchors), the same model will be applicable to active loci generally. Indeed, we have shown that these predictive heteromorphic polymer simulations can be successfully applied to loci in different cell types and organisms, such as α and β-globin or *SOX2*, in stem cells and tissue-derived cell lines, and in human or mouse. Unlike other approaches that require 3C-based data as an input, the HiP-HoP model does not require these data, making it well suited for predicting chromatin structure at promoters and enhancers genome-wide.

## STAR★Methods

### Key Resources Table

**Table:** 

REAGENT or RESOURCE	SOURCE	IDENTIFIER
**Antibodies**		

H3K27ac (Anti-Histone H3 Acetyl K27)	Abcam	Cat# ab4729; RRID: AB_2118291
Anti-Rad21, Rabbit polyclonal	Abcam	Cat# ab992; RRID: AB_2176601
CTCF (D31H2) XP Rabbit mAb antibody	Cell Signaling Technology	Cat# 3418; RRID: AB_2086791

**Bacterial and Virus Strains**		

Fosmid WIBR1-0075F18: Rcn1 gene FISH probe	BACPAC resource	ID: WIBR1-0075F18
Fosmid WIBR1-0322P22: Pax6 gene FISH probe	BACPAC resource	ID: WIBR1-0322P22
Fosmid WIBR1-1660D19: Elp4 gene FISH probe	BACPAC resource	ID: WIBR1-1660D19
Fosmid WIBR1-2859L14: DRR FISH probe	BACPAC resource	ID: WIBR1-2859L14
Fosmid WIBR1-1230I10: URR FISH probe	BACPAC resource	ID: WIBR1-1230I10

**Chemicals, Peptides, and Recombinant Proteins**		

Formaldehyde solution 37%	Sigma	Cat# 252549

**Critical Commercial Assays**		

NebNext DNA Library Prep Kit	NEB	Cat# E6040
NEBNext mRNA Library Prep Master Mix Set	NEB	Cat# E6110
Protein G Dyna beads	Thermo Fisher Scientific	Cat# 10003D
RiboMinus Eukaryote Kit for RNA-Seq	Thermo Fisher Scientific	Cat# A1083708
SYBR Green I Nucleic Acid Gel Stain	Thermo Fisher Scientific	Cat# S7563
Tapesation D1000 ScreenTapes	Agilent	Cat# 5067-5582
Tapesation D1000 Reagents	Agilent	Cat# 5067-5583
NebNext Multiplex Oligos for Illumina	NEB	Cat# E7335
Nextera DNA Lib prep kit: Tn5 Transposes, TD buffer	Illumina	Cat# FC-121-1030
GenomePlex WGA2 Kit	Sigma	Cat# WGA1-50RXN
GenomePlex WGA2 Reamplification Kit	Sigma	Cat# WGA3-50RXN
CYTAG CGH labeling kit for oligo arrays	Enzo	Cat# ENZ-42671
NimbleGen Dual-Color Labeling Kit	Roche	Cat# 06370250001
NimbleGen Hybridization and Sample Tracking Control Kit	Roche	Cat# 05993776001
NimbleGen Wash Buffer Kit,	Roche	Cat# 0558450700
SeqCap EZ HE-Oligo Kit A	Roche	Cat# 06777287001
SeqCap EZ Hybridization and Wash Kit	Roche	Cat# 05 634 261 001
SeqCap EZ Pure Capture Bead Kit	Roche	Cat# 06 977 952 001
SeqCap EZ Accessory Kit	Roche	Cat# 07145594001

**Deposited Data**		

RNA-seq data: β-TC3, MV+, RAG cells	This paper	GEO: GSE119660
ATAC-seq data: β-TC3, MV+, RAG cells	This paper	GEO: GSE119656
ChIP-Chip Data: Nimblegen 720K, H3K27ac	This paper	GEO: GSE119659
ChIP-Chip Data: Nimblegen 720K, CTCF, Rad21	This paper	GEO: GSE119658
ChIP-Chiip Data: Agilent 180k, Rad21	This paper	GEO: GSE120665
NG-Capture-C Data: β-TC3, MV+, RAG cells	This paper	GEO: GSE120666
The processed simulation and experimental data used to generate all figures. The full set of 200 simulated locus configurations for each cell type used to generate plots and simulation snap-shot images for Figures 4 and 5, and Videos S1, S2, S3, S4, and S5. An input script and python driver script along with example initialization configurations which can be used to run a HiP-HoP simulation of the Pax6-HIGH cells using the LAMMPS software.	This paper	Edinburgh DataShare https://datashare.is.ed.ac.uk/handle/10283/3178

**Experimental Models: Cell Lines**		

MV+, cells, *Mus musculus*, lens epithelium	Originally supplied by Dr. Alan Prescott. Derived from cultured lens epithelia from a wildtype C57BL6 mouse. McBride et al., 2011	N/A
RAG cells, *Mus musculus*, kidney carcinoma	ATCC	Cat# CCL-142, RRID: CVCL_3575
β-TC3 cells, *Mus musculus*, insulinoma	DSMZ	Cat# ACC-324, RRID: CVCL_0172

**Oligonucleotides**		

5′ Biotin Ultramer Capture Oligo	IDT	See Table S1

**Software and Algorithms**		

LAMMPS: Simulations work was performed using the LAMMPS molecular dynamics software	Plimpton, 1995	https://lammps.sandia.gov/
Additional simulation scripts	This paper; Edinburgh DataShare	https://datashare.is.ed.ac.uk/handle/10283/3178
Ringo: Microarray data were processed using the open source R package Ringo, Bioconductor	Toedling et al., 2007	https://doi.org/10.18129/B9.bioc.Ringo
Bowtie2	Langmead and Salzberg, 2012	http://bowtie-bio.sourceforge.net/bowtie2/index.shtml
SAMtools	Li et al., 2009	http://samtools.sourceforge.net/
Trim Galore		https://www.bioinformatics.babraham.ac.uk/projects/trim_galore/
BEDtools	Quinlan and Hall, 2010	https://bedtools.readthedocs.io/en/latest/
MACS2	Zhang et al., 2008	https://github.com/taoliu/MACS
STORM	Schones et al., 2007	http://rulai.cshl.edu/storm
Subread feature counts	Liao et al., 2014	http://subread.sourceforge.net/

**Other**		

HEK293T Single-cell transcription data	Darmanis et al., 2015	GEO: GSE67835
H1-hESC Single-cell transcription data	Leng et al., 2015	GEO: GSE64016

### Contact for Reagent and Resource Sharing

Further information and requests for resources and reagents should be directed to and will be fulfilled by the Lead Contact, Nick Gilbert (nick.gilbert@ed.ac.uk).

### Experimental Model and Subject Details

#### Cell Types

Pax6-HIGH cells (also known as β-TC3 cells) were isolated from a mouse insulinoma (Henseleit et al., 2005), Pax6-ON cells (also known as MV+ cells) were derived from cultured mouse lens epithelia (McBride et al., 2011), and Pax6-OFF cells (also known as RAG cells) were derived from a renal adenocarcinoma from BALB/c strain, and purchased from ATCC (no. CCL-142). All cell lines were cultured in Dulbecco’s Modified Eagle Medium (DMEM) (ThermoFisher) supplemented with 10% fetal calf serum and 1% Penicillin-Streptomycin at 37°C in 5% CO_2_. No cell authentication was performed, and sex of cell line is not known.

### Method Details

#### ChIP-chip

H3K27ac (Abcam, ab4729), CTCF (Cell signaling, D31H2 XP Rabbit mAb #3418), and Rad21 (Abcam Ab992) antibodies where used for ChIP with two biological replicates per antibody condition. Two T75 flasks of 80% confluent cells were used for each ChIP, representing two biological replicates per antibody condition and this was performed using a cross-linked ChIP protocol adapted from (Creyghton et al., 2010). All centrifugation steps were performed at 1200 g for 5 min at 4°C. Culture media was aspirated and replaced with 20 mL of fresh DMEM (ThermoFisher) with no additives. 550 μL of 37% Formaldehyde (Sigma) was added for 10 min, and quenched with 2 mL of 2 M glycine solution at 4°C for 5 min. Flasks were washed with PBS twice at 4°C, and a cell scrapper was used to remove the adherent cell layer in 10 mL cold PBS. Fixed cells were centrifuged, supernatant removed, and resuspended in 3 mL of Cell Lysis Buffer 4°C for 10 min. Nuclei where washed with 5 mL Pre ChIP Wash buffer, centrifuged, and resuspended in 2.5 mL of Sonication buffer. Samples were sonicated on ice for 13 min, 30 s on, 30 s off at 50% amplitude with a Soniprep 150 probe sonicator. DNA was checked on an agarose gel to achieve an ideal DNA size of ∼300-500 bp. All samples were aliquoted (500 μl) and stored at −80°C.

Each IP was performed with 25 μl of Protein G Dyna Beads (ThermoFisher), pre-washed with 2x 1 mL PBS/BSA, and incubated in 200 μl PBS/BSA with 10 μl of capture antibody (CTCF antibody (D31H2 XP Rabbit mAb #3418), Rad21 antibody (Abcam Ab992), H3K27ac (Abcam, ab4729) or control Rabbit IgG (I5006 Sigma)) for 3 hr, rotating at 4°C. One T75 worth of cross-linked and sonicated chromatin was used per ChIP, diluted in sonication buffer and pre-blocked using 10 μl of native beads for 30 min rotating at 4°C. Blocking beads were removed and added to pre-prepared bead/antibody complex, for incubation overnight rotating at 4°C. 10% of chromatin was stored as input. CTCF and Rad21 Beads were washed sequentially at 4°C in 1 mL each of ChIP wash, 1x ChIP wash 1, 3x ChIP wash 2, 1x ChIP wash 3, beads were transferred to a new tube, and washed 2x ChIP wash 4, 10 min 4°C (Table S1). H3K27ac ChIP was were washed 5x RIPA Buffer B, and 2x ChIP wash 4. All chromatin was eluted from beads in 400 μl elution buffer, shaking at 65°C overnight along with the 10% Input sample to reverse the cross-linking. DNA was treated with RNaseA (0.2 μg/μl final) for 2 hr at 37°C, and proteinase K (0.2 μg/μl final) for 2 hr at 50°C. Two standard phenol chloroform extractions were performed with 2 mL Phase lock tubes (5 prime). The resulting DNA was purified using a PCR clean up kit (QIAGEN), with a double 50 μl final elution and stored at −20°C.

#### ChIP Solutions

ChIP Lysis Buffer: 50 mM HEPES pH 6.8, 140 mM NaCl, 10% (v/v) glycerol, 0.5% (v/v) IPEGAL, 0.25% (v/v) Triton X, 1mM EDTA, 0.25 mM PMSF
Pre ChIP Wash: 200 mM NaCl, 10 mM Tris pH 8, 1 mM EDTA, 0.5 mM EGTA, 0.25 mM PMSF
Sonication Buffer: 100 mM NaCl, 0.1% (v/v) Na deoxycholate, 0.5% N-lauroylsarcosine 10 mM Tris pH8, 1 mM EDTA, 0.5 mM EGTA, 0.25 mM PMSF
ChIP Wash 1: 25 mM Tris pH 8, 150 mM NaCl, 1% Triton X-100, 0.1% SDS, 2 mM EDTA, 0.25 mM PMSF
ChIP Wash Buffer 2 (high salt): 25 mM Tris pH 8, 500 mM NaCl, 1% Triton X-100, 0.1% SDS, 2 mM EDTA, 0.25 mM PMSF
ChIP Wash Buffer 3 (detergent): 10 mM Tris pH8, 250 mM LiCl, 1% Triton X-100, 1% IPEGAL, 1% sodium deoxycholate, 2 mM EDTA, 0.25 mM PMSF
Elution Buffer: 50 mM Tris pH 8, 10 mM EDTA, 1% SDS
RIPA Buffer B: 50 mM HEPES (pH 7.6), 1 mM EDTA, 0.7% Na deoxycholate (v/v), 1% (v/v) Nonidet P-40, and 0.5 M LiCl
ChIP Wash 4: TE, 50 mM NaCl

#### Array Hybridization

CTCF, Rad21, and H3K27ac ChIP and Input samples where whole genome amplified (GenomePlex Whole Genome Amplification Kit, Sigma), Rad21 and Inputs had a second amplification (GenomePlex Whole Genome Reamplification Kit, Sigma) purified (QIAquick, QIAGEN) and labeled (NimbleGen Dual-Color Labeling Kit, Roche Cat. 06370250001). ChIP samples (Cy5) and Input samples (Cy3) were hybridized using a NimbleGen Hybridization and Sample Tracking Control Kit (Roche Cat. 05993776001) according to the manufacturer’s instructions. Slides were washed (NimbleGen Wash Buffer Kit, Roche Cat. 0558450700) and scanned at 2 μm resolution on a MS 200 Microarray Scanner (Nimblegen). Images were processed using NimbleScan (version 2.5). Two replicates of ChIP hybridization along with input to custom genomic microarrays (Nimblegen 720K) tiling a 66 Mb region around *Pax6* (Chr2:75,000,000-141,000,000) were performed. Agilent 180K custom tiling arrays were used for Rad21 ChIP-Chip samples (4 Mb *Pax6*), labeling was performed with CYTAG CGH labeling kit (Enzo) and processed at the VU microarray facility, Amsterdam. For ChIP-ChIP Ringo (Bioconductor) was used for pre-processing, normalization, combining replicates and peak calling of ChIP-chip data (Toedling et al., 2007).

##### CTCF Site Motif Directionality

STORM software (Schones et al., 2007) was used to search the sequence under each CTCF peak for the consensus motif reported in Kim et al. (2007). The width of the CTCF peaks in our ChIP-on-chip data were typically 3 kbp, so for each a 3 kbp window around the peak center was searched. It was possible that the peak could contain more than one instance of the motif, and some peaks contained a motif on both strands. In this case, the motif which best matched the consensus motif was chosen, unless the match was less than 2% better, in which case the site was designated as having both directions.

#### ATAC-Seq

ATAC-seq was performed in duplicate for Pax6-HIGH, ON and OFF cells. Cells were cultured and harvested to provide a single cell suspension. To make nuclei cells were pelleted at 1400 rpm and resuspend in 2ml cold NBA buffer (85 mM NaCl, 5.5% sucrose, 10 mM Tris-HCl pH 7.5, 0.2 mM EDTA, 0.2 mM PMSF, 1 mM DTT, 1 × Protease Inhibitors) and mixed with 2 mL of cold NBB buffer (for MV+, NBA buffer + 0.2% NP40; β-TC3 and RAG, NBA buffer + 0.1% NP40) for 2 min at 4°C, and centrifuged at 2000 rpm before washing in 2 mL NBR 4°C, centrifuged again and resuspended in 1ml NBR (85 mM NaCl, 5.5% sucrose, 10 mM Tris-HCl pH 7.5, 3 mM MgCl_2_, 1.5 mM CaCl_2_, 0.2 mM PMSF, 1 mM DTT). Nuclei where checked for purity, and counted. Protocol for transposition reaction and PCR based on Buenrostro et al. (2013). 50,000 nuclei per condition where pelleted and resuspended in 1 × TD Buffer (Nextera, Illumina) with 2.5 μl Tn5 transposase (Nextera, Illumina) in 50 μl volume, at 37°C 300rpm for 30 min. DNA was purified by MiniElute PCR purification (QIAGEN), before test amplification to calculate library amplification cycle number with NEBNext Ultra II Q5 Master Mix (NEB), Sybr green (ThermoFisher) and customized Nextera PCR Primers (Buenrostro et al., 2013) using a LightCycler480 (Roche). ATAC-seq libraries where uniquely indexed with customized Nextera PCR Primers and amplified with 11 cycles of amplification before QIAquick PCR purification (QIAGEN), and Ampure XP Bead (Beckman Coulter) size selection, quality controlled, and quantified on a D1000 Tapestation Screentape (Agilent), and sequenced on an Illumina HiSeq 4000 75bp PE sequencing. Sequenced reads where trimmed for Nextera adapters using Trim galore and aligned to the mm9 genome with bowtie2 (Langmead and Salzberg, 2012), and read pile-ups generated and corrected for read depth using the Bedtools “genome coverage” tool. Locus specific peak calling was performed for the *Pax6* region and genome-wide peak calling was performed using MACS version 2.1.1, with a q-value cut off pf 0.05.

#### RNA-Seq

Experiments were performed and analyzed (Buckle et al., 2018), with three experimental replicates for Pax6-HIGH, ON and OFF cells (data from GSE116811). Briefly, total RNA was extracted using QIAGEN RNeasy mini kit (QIAGEN) and ribosomal RNA depleted using RiboMinus Eukaryote Kit for RNA-Seq (Life Technology); libraries were made with NEBNext mRNA Library Prep Master Mix Set (NEB) and sequenced on an Illumina Hi-Seq 2000 SE 50. Reads where aligned to the mm9 genome using TopHat v2 and processed with Samtools v1.6 (Li et al., 2009), and the Bedtools “genome coverage” tool (Quinlan and Hall, 2010). Aligned BAM files were processed with the Subread v1.5 “feature counts” tool (Liao et al., 2014) to generate FPKM scores against mm9 RefSeq genes. Single cell RNA-seq data were used: HEK293T (GSE49321) or Human H1-hESCs (GSE64016).

#### NG Capture-C

NG Capture-C was performed on Pax6-HIGH, ON and OFF cells (Davies et al., 2016, Hughes et al., 2014), with the following alterations. Two replicates of $5\times 10^{6}$ cells where processed for each cell type, fixed with 2% formaldehyde, and lysed for 15 min with standard 3C lysis buffer before snap freezing. Cells were further lysed by re-suspension in water, and then in 0.5% SDS at 62°C for 10 min. Each replicate was split between three tubes, re-suspended in 800 μL 1 × *Dpn*II buffer (NEB) with 1.6% Triton X-100, and digested with 3 sequential additions of 750 units *Dpn*II enzyme at 37°C with shaking 1200rpm over 24 hr. Samples were heat inactivated at 65°C for 20 min, and 3 samples from each replicate combined into 7 mL with 1 × T4 DNA Ligase Buffer (NEB), with 1% Triton X-100, and 12,000 units of T4 DNA ligase at 16°C overnight. Samples were treated with Proteinase K at 65°C overnight and RNase A/T1 (ThermoFisher) at 37°C for 1 hr, before a standard Phenol/Chloroform, Chloroform extraction and ethanol precipitation was performed. Complete digestion and ligation was assessed by gel electrophoresis.

Purified 3C DNA from each sample was sonicated to 200-400 bp with a Soniprep 150 probe sonicator at 4°C and purified with a standard Ampure XP Bead protocol (Beckman Coulter) using a 1/1.5 DNA to bead ratio. Two Illumina sequencing libraries were prepared per capture pool replicate, with 6 μg of starting DNA in each, and generated using NEBNext DNA Library Prep Kit (NEB), with samples indexed with unique barcodes using NEBNext Multiplex Oligos for Illumina (NEB). Two separate capture pools were designed to the following *Pax6* locus elements: CTCF and/or Cohesin binding sites, known and predicted enhancer clusters, and the multiple promoters of three genes in the locus *Pax6*, *Elp4* and *Rcn1* (a full list of targeted restriction enzyme fragments is given below). Capture oligos were designed to each end of the targeted *Dpn*II fragments (Davies et al., 2016), and each synthesized as a separate 4 nM synthesis, with a 5′-biotin label on a 120 bp Ultramer (IDT) (Table S1). All capture oligos from each of the two capture pools were mixed at equimolar amounts and pooled to a final concentration of 13 pmol in a volume of 4.5 μL per sequence capture. Libraries where sized and quality controlled on a D1000 Tapestation tape (Agilent).

NG Capture-C sequence capture was performed using SeqCap EZ HE-Oligo Kit A or B (dependent on the multiplex barcode) and SeqCap EZ Accessory Kit (Nimblegen) (Davies et al., 2016), using each of the two capture pools, with 1.5-2 μg 3C library DNA per hybridization reaction. Each hybridization reaction was performed on a thermocycler at 47°C and incubated for between 66 and 72 hr. Each hybridization reaction was then bound to streptavidin beads from SeqCap EZ Pure Capture Bead Kit and washed with SeqCap EZ Hybridization and Wash Kit (Nimblegen), following the manufacture’s protocol. Hybridization reactions were split into two and libraries re-amplified using Post LM-PCR oligos (Nimblegen) and Q5 High-Fidelity DNA polymerase (NEB) directly from the beads, and then the DNA was purified using Ampure XP Bead 1/1.8 DNA to bead ratio. A second hybridization reaction was performed as above on the re-amplifed 3C libraries with two reactions pooled together (∼1 μg in each) and incubated for 22-24 hr. Washed and re-amplified double captured libraries where sized and quality controlled on a D1000 Tapestation tape (Agilent), and paired-end sequenced on an Illumina Hi-seq 2500 or Hi-seq 4000.

##### Data Analysis

Capture-C data were analyzed according to methods developed by Davies et al. (2016) and Hughes et al. (2014). First, paired end reads were tested for overlaps, and if necessary combined into single reads. Reads were “*in silico* digested,” and broken into shorter fragments at *Dpn*II sites. Thus, each paired end read was converted into a set of *Dpn*II fragments, which were called a read group; each fragment was then aligned separately to the mm9 reference genome as single end reads using Bowtie (Langmead and Salzberg, 2012). The resulting mapped fragments were recombined into their read groups, and duplicates were removed (duplicates are defined as two read groups where the exact same fragments appear in the same order within the read, including those which could not be mapped to the genome; such duplicates arise due to PCR artifacts). Each mapped fragment was expanded to the *Dpn*II fragment from which it originated, and only read groups containing a targeted *Dpn*II fragment were retained. Read groups containing multiple targeted *Dpn*II fragments were also removed: since the relative efficiency of the oligo capture for each target is not known, these reads are not quantitative. Read groups containing exactly one targeted *Dpn*II fragment, and only one mapped “reporter” fragment were retained as “informative reads.” Since *Dpn*II sites are only digested with a finite probability, we further discarded read groups which showed an interaction between a target fragment and a fragment within a 500-bp exclusion region around another target fragment (these may have been “double captured,” and so again are not quantitative).

The above scheme gives an interaction frequency between each target *Dpn*II fragment, and every other mappable *Dpn*II fragment in the genome. We normalized such that the total number of interactions for each target was 100,000,000 reads genome-wide; this implies the assumption that each target should have the same interaction “visibility.” To obtain the interaction profiles shown in figures, we applied a sliding binning window, which collects the data into 3-kbp bins which contain data from a 6-kbp window (Figures S2B–S2D have 25-kbp bins which contain data from 50-kbp windows).

To assess “How much do the *Pax6* promoters interact with the upstream regulatory region (URR)”?, as shown in Figure 2B, we took the data for the probes at *Pax6* (probes Pax6_P0, Pax6_P1, and Pax6_Pα), and counted the number of normalized reads falling within a window of 10 kbp around the probe at the URR (probe CTCF6).

In Figure S2E we show a measure of the “directionality” of interaction for each probe. This is defined by counting and checking the direction of the interactions within a 2 Mbp window around the probe; specifically

$$\text{directionality}=\frac{N_{\text{ds}}-N_{\text{us}}}{N_{\text{ds}}+N_{\text{us}}}$$

where $N_{\text{us}}$ and $N_{\text{ds}}$ are the number of normalized reads which show interactions with regions upstream and downstream of the probe respectively.

#### Capture-C Probes

List of the targeted restriction enzyme fragments, names, which pool of oligos they belong to, and genomic position (mm9 genome build).

Target name pool restriction fragment
Pax6_P1 1 chr2:105515339-105515903
Pax6_P0 1 chr2:105508737-105509119
Pax6_Palpha 1 chr2:105521487-105522094
CTCFp11 1 chr2:105511309-105512303
7CE12_CTCF 1 chr2:105527664-105528138
Elp4_pro 1 chr2:105744078-105745820
CTCF5 2 chr2:105639621-105640392
CTCF6 2 chr2:105456258-105457375
CTCF4 2 chr2:105748530-105748848
CTCF7 2 chr2:105363080-105364092
CTCF10B 2 chr2:105173505-105175699
Rcn1_pro 2 chr2:105238728-105239162
e200_Enh 2 chr2:105284625-105285412

#### Three-Dimensional DNA Fluorescence *In Situ* Hybridization

Cells were grown overnight on glass slides. Slides were rinsed with PBS and fixed in 4% paraformaldehyde for 10 min (Naughton et al., 2013), rinsed with PBS and cells were permeabilized for 10 min on ice with PBS supplemented with 0.5% Triton X-100. After rinsing, slides were air-dried and stored at −70°C. For processing, slides were washed briefly with PBS and incubated with 2 × SSC supplemented with 100 μg ml^−1^ RNase A (Invitrogen) at 37°C for 60 min. Slides were then rinsed briefly with 2 × SSC, dehydrated through an ethanol series and air-dried. Slides were warmed by incubation in a 70°C oven for 5 min before denaturation for 40 min in 70% formamide in 2 × SSC, pH 7.5, at 80°C. Slides were then transferred to 70% ethanol on ice, dehydrated through an ethanol series and air-dried before overnight hybridization at 37°C with probes. Fosmid probes (BacPac resources) were labeled in green-500-dUTP (ENZO life sciences), digoxigenin-11-UTP (Roche) or biotin-16-dUTP (Roche). 100 ng of each labeled probe was hybridized with 5 μg salmon sperm and 20 μg human Cot1 DNA. Slides were washed four times for 3 min in 2 × SSC at 45°C and four times for 3 min in 0.1 × SSC at 60°C before being transferred to 4 × SSC with 0.1% Tween 20 at room temperature. Probes used in this study are listed in the table below. Digoxigenin-labeled probes were detected by using one layer of rhodamine-conjugated sheep anti-digoxigenin and a second layer of Texas red–conjugated anti-sheep (Vector Laboratories). Biotin-labeled probes were detected by using one layer of Cy5-conjugated streptavidin followed by a layer of biotin-conjugated anti-avidin and a second layer of Cy5-conjugated streptavidin (Vector Laboratories). Slides were counterstained with 0.5 μg ml^−1^ DAPI and mounted.

Four-color stained 3D slides were imaged using a Photometrics Coolsnap HQ2 CCD camera (Photometrics Ltd, Tucson, AZ), on a Zeiss Axioskop II MOT fluorescence microscope with Plan-neofluar or Plan apochromat objectives, a Lumen 200W metal halide light source (Prior Scientific Instruments, Cambridge, UK) and Chroma #89000ET single excitation and emission filters (Chroma Technology, Rockingham, VT) with the excitation and emission filters installed in Prior motorised filter wheels. A piezoelectrically driven objective mount (PIFOC model P-721, Physik Instrumente GmbH & Co, Karlsruhe) was used to control movement in the z dimension. Hardware control, image capture and analysis were performed using Volocity (Perkinelmer Inc, Waltham, MA). Image stacks (0.2 μm slices) were collected from at least 70 randomly selected nuclei for each experiment. Images were deconvolved using a calculated PSF in Volocity (Perkinelmer Inc, Waltham MA) and the distances between probes was measured using Volocity. Image color balance was adjusted to improve data visualization in the manuscript.

##### Additional Quantities from Three-Color FISH Measurements

The three-color probes allow simultaneous measurement of three distances in a single cell. This information was used to give a measure of the size of the locus, S, for each cell, defined as

$$S=\frac{1}{3}\sqrt{d_{1}^{2}+d_{2}^{2}+d_{3}^{3}}$$

where $d_{\alpha }$ are the separations between pairs of probes. This is equivalent to finding the radius of gyration of the three probe centers. The distributions of S for each cell type are given in Figure 2.

As well as a size of the locus, this triplet of separations can be used to give a measure of the cell-to-cell variability of the conformation of the locus. The three separations can be thought of as a vector representing a point in a three-dimensional “conformation” space. The set of imaged cells gave a set of points in the conformation space (Figure S3E), and the volume of the region which is taken up by this cloud of points was used as a measure of the level of cell-to-cell variation of conformations within that population. To quantify this volume we found the gyration tensor, defined as

$$G_{\alpha \beta }=\frac{1}{2N^{2}}\sum_{i=1}^{N}\sum_{j=1}^{N}\left(d_{\alpha }^{\left(i\right)}-d_{\alpha }^{\left(j\right)}\right)\left(d_{\beta }^{\left(i\right)}-d_{\beta }^{\left(j\right)}\right)$$

where the sums of indices i and j run over the N imaged cells, and $d_{\alpha }^{\left(i\right)}$ is the separation between pair of probes α in cell i. The three principal moments of this tensor give the dimensions of an ellipsoid representing the cloud of points, and we took the product of these as a measure of its volume, or the cell-to-cell variability of the locus conformation.

#### FISH Probes Used in This Study, Including Genomic Position (mm9)

Name Fosmid ID genome position Label
Rcn1 WIBR1-0075F18 chr2:105218785-105254871 green-500-dUTP
Pax6 WIBR1-0322P22 chr2:105508853-105550057 digoxigenin-11-dUTP
Elp4 WIBR1-1660D19 chr2:105726249-105764673 biotin-16-dUTP
URR WIBR1-1230I10 chr2:105430492-105469674 digoxigenin-11-dUTP
Pax6 WIBR1-0322P22 chr2:105508853-105550057 biotin-16-dUTP
DRR WIBR1-2859L14 chr2:105612707-105655961 green-500-dUTP

#### Polymer Simulations

Coarse grained molecular dynamics simulations were performed, in which collections of molecules are represented by “beads,” which interact with phenomenological force fields and move according to Newton’s laws. A chain of beads connected by springs represent a region of the chromosome, and individual beads represent complexes of transcription factors or other DNA binding proteins (TFs). We also include loop extruding factors, which are represented by additional springs between non-adjacent chromatin chain beads. We use the multi-purpose molecular dynamics package LAMMPS (Large-scale Atomic/Molecular Massively Parallel Simulator) (Plimpton, 1995).

##### Simulation Method

The position of the i th bead in the system, representing a chromatin bead or a TF, evolves according to the Langevin equation

(1) $$m_{i}\frac{d^{2}r_{i}}{dt^{2}}=-\nabla U_{i}-\gamma_{i}\frac{dr_{i}}{dt}+\sqrt{2k_{B}T\gamma_{i}}\eta_{i}\left(t\right),$$

where $r_{i}$ is the position of bead i with mass $m_{i}$, $\gamma_{i}$ is the friction due to an implied solvent, and $\eta_{i}$ is a vector representing random uncorrelated noise such that

$$\langle \eta_{\alpha }\left(t\right)\rangle =0\;\text{and}\;\langle \eta_{\alpha }\left(t\right)\eta_{\beta }\left(t^{\prime }\right)\rangle =\delta_{\alpha \beta }\delta \left(t-t^{\prime }\right).$$

The noise is scaled by the energy of the system, given by the Boltzmann factor $k_{B}$ multiplied by the temperature of the system T, taken to be 310 K. The potential $U_{i}$ is a sum of interactions between bead i and all other beads, and we use phenomenological interaction potentials as shown below. Equation 1 is solved in LAMMPS using a standard Velocity-Verlet algorithm.

For the chromatin fiber the i th bead in the chain is connected to the i+1 th with a finitely extensible non-linear elastic (FENE) spring given by the potential

(2) $$U_{\text{FENE}}\left(r_{i,i+1}\right)=U_{\text{WCA}}\left(r_{i,i+1}\right)-\frac{K_{\text{FENE}}R_{0}^{2}}{2}\log\left[1-{\left(\frac{r_{i,i+1}}{R_{0}}\right)}^{2}\right],$$

where $r_{i,i+1}=\left|r_{i}-r_{i+1}\right|$ is the separation of the beads, and the first term is the Weeks-Chandler-Andersen (WCA) potential

(3) $$\frac{U_{\text{WCA}}\left(r_{ij}\right)}{k_{B}T}=\left\{ \begin{array}{c} 4\left[{\left(\frac{d_{ij}}{r_{ij}}\right)}^{12}-{\left(\frac{d_{ij}}{r_{ij}}\right)}^{6}\right]+1,\;r_{ij}<2^{1/6}d_{ij}\\ 0,\;\text{otherwise}, \end{array}\right.$$

which represents a steric interaction that prevents adjacent beads from overlapping; here $d_{ij}$ is the mean of the diameters of beads i and j. The diameter of the chromatin beads is a natural length scale with which to parametrize the system; we denote this σ, and use this to define all other length scales. The second term in Equation 2 gives the maximum extension of the bond, $R_{0}$; throughout we use $R_{0}=1.6\sigma$, and set the bond energy $K_{\text{FENE}}=30k_{B}T$. The bending rigidity of the polymer is introduced via a Kratky-Porod potential for every three adjacent chromatin beads

$$U_{\text{BEND}}\left(\theta \right)=K_{\text{BEND}}\left[1-\cos\left(\theta \right)\right],$$

where θ is the angle between the three beads as given by

$$\cos\left(\theta \right)=\frac{\left[r_{i}-r_{i-1}\right]\cdot \left[r_{i+1}-r_{i}\right]}{\left|r_{i}-r_{i-1}\right|\left|r_{i+1}-r_{i}\right|},$$

and $K_{\text{BEND}}$ is the bending energy. The persistence length in units of σ is given by $l_{p}=K_{\text{BEND}}/k_{B}T$. Finally, steric interactions between non-adjacent chromatin beads are also given by the WCA potential (Equation 3).

Each TF is represented by a single bead and the WCA potential is used to give a steric interaction between these. Chromatin beads are labeled as binding or not-binding according to the input data (see below). For the interaction between proteins and the chromatin beads labeled as binding, we use a shifted, truncated Lennard-Jones potential

$$U_{\text{LJcut}}\left(r_{ij}\right)=\{\begin{array}{c} U_{\text{LJ}0}\left(r_{ij}\right)-U_{\mathrm{LJ}0}\left(r_{\text{cut}}\right),r_{ij}<r_{\text{cut}}\\ 0,\text{otherwise}, \end{array}$$

with

$$U_{\text{LJ}0}\left(r\right)=4\epsilon \left[{\left(\frac{d_{ij}}{r}\right)}^{12}-{\left(\frac{d_{ij}}{r}\right)}^{6}\right],$$

where $r_{\text{cut}}$ is a cut off distance, and $r_{ij}$ and $d_{ij}$ are the separation and mean diameter of the two beads respectively. For simplicity we set the diameter of the protein complexes equal to that of the chromatin beads, $d_{ij}=\sigma$. This potential leads to an attraction between a protein and a chromatin bead if their centers are within a distance between $2^{1/6}\sigma$ and $r_{\text{cut}}$. Here ϵ is an energy scale, and we set $\epsilon =8k_{B}T$ and $r_{\text{cut}}=1.8$ for attractive interactions between TFs and binding chromatin beads. To model non-specific interactions, TFs also have a weak attraction $\left(\epsilon =2k_{B}T\right)$ with non-binding chromatin beads. Throughout the simulation the TFs switch back and forward between a binding and a non-binding state with rate $k_{\text{sw}}$; when in the latter state, interactions with chromatin beads revert to the WCA potential. As detailed in (Brackley et al., 2017), this non-equilibrium switching allows the formation of stable protein clusters, where the constituents both bind stably, and turn over quickly.

Loop extruding factors (LEs) are represented by additional springs between non-adjacent beads along the chromatin chain. Binding of extruding factors occurs at a rate $k_{\text{on}}$, and we choose at random a pair of i,i+2 beads from the chromatin – a spring bond is added between the pair. We use a harmonic spring with WCA short range repulsion given by the potential

$$U_{\text{EXTR}}\left(r_{i,j}\right)=U_{\text{WCA}}\left(r_{i,j}\right)+K_{\text{EXTR}}{\left(r_{i,j}-r_{0}\right)}^{2},$$

where $r_{i,j}=\left|r_{i}-r_{j}\right|$ is the separation of the beads, $K_{\text{EXTR}}$ is the bond energy, and $r_{0}$ is the bond length; we set $K_{\text{EXTR}}=40k_{B}T$ and $r_{0}=1.5\sigma$. To simulate extrusion, at regular time intervals (rate $k_{\text{ex}}$) we move this spring to the next pair of beads, i.e., i,i+2→i−1,i+3→i−2,i+4 etc. LEs are removed from the chromatin with rate $k_{\text{off}}$. We keep the number of extruders present in the system fixed, so that at any point in time each extruder can be in a bound or unbound state. LEs cannot move past each other, and the movement halts if blocked by another LE. Additionally, if an LE reaches a correctly oriented “loop anchor” bead, movement of that side of the spring stops (the other side keeps moving unless it also meets a correctly oriented loop anchor); the unbinding dynamics are not affected by extrusion halting at loop anchors. Loop anchors and their direction are defined according to CTCF binding data (detailed below). The LE dynamics are performed using a python script which drives the LAMMPS library.

The polymer is initialized as a random walk, and the dynamics are first evolved in the absence of TF interactions and extruders in order to generate an equilibrium coil conformation. Interactions with the TFs and extruders are then switched on, and the dynamics are evolved until a new steady-state conformation is obtained.

##### Simulation Units and Parameters

So far the system has been described in units σ, m, and $k_{B}T$ for lengths, masses, and energies respectively. Another important parameter is the simulation resolution, or bead size in bp, which, unless otherwise stated, we fix at 1 kbp per bead. Since the packaging of DNA into chromatin is not fully understood, we do not fix the physical value of the length unit σ ahead of running the simulations. Instead, we perform the simulations, and compare with the FISH data, using that to estimate the physical value of σ – see below for details (note that this value does not affect the simulated Capture-C or simulated Hi-C results).

As regards timescales, we first note that the previously defined simulation units define a natural time unit $\tau_{\text{LJ}}=\sqrt{\sigma^{2}m/k_{B}T}$. Another important timescale is the Brownian time $\tau_{\text{B}}=\sigma^{2}/D_{i}$ (the time it takes for a bead to diffuse across its own diameter σ), and it is this which we use to determine the mapping of simulation time to real time. Here $D_{i}$ is the diffusion constant for bead i, and is related to the friction via the Einstein relation $D_{i}=k_{B}T/\gamma_{i}$. For simplicity we take all beads (TF and polymer beads) to have the same diffusion constant and mass m=1, and set $\gamma_{i}=2$ so that $\tau_{\text{B}}=2\tau_{\text{LJ}}$. This means the system is overdamped, as should be the case physically; beads have more “inertia” than in reality (this is necessary to keep overall simulation times manageable), but this will only affect very early times, whereas we examine locus configurations at steady state. To map to real time we measure the mean squared displacement (MSD) for all polymer beads (this depends on polymer density, binding TFs and LEs, so can vary across cell types and with other simulation details – for each simulation model we take an average across cell types). We then find the value of $\tau_{\text{B}}$ which gives the best fit to experimental results from Hajjoul et al. (2013), who measured the MSD for various chromatin loci in live yeast cells.

Other important parameters are: the TF switching rate, chosen as $k_{\text{sw}}=1.25\times 10^{-4}\tau_{\text{LJ}}^{-1}$, and the LE unbinding rate and extrusion velocity, chosen as $k_{\text{off}}=2.5\times 10^{-5}\tau_{\text{LJ}}^{-1}$ and $k_{\text{ex}}=2\;\text{bp}\;\tau_{\text{LJ}}^{-1}$ respectively. The 5000 bead polymer is in a periodic simulation box of side 150σ (meaning it is in the dilute regime, though the mapping of the timescales via the MSD means that macromolecular crowding is effectively taken into account), and there are 4000 TFs. The persistence length of the polymer is set at $l_{p}=4\sigma$ (though see below). Simulation results presented in the main figures are taken from 10 independent simulations, each run for $50\times 10^{4}\tau_{\text{LJ}}$ (after TFs and LEs are switched on). Equation 1 is integrated with a constant time step $\text{Δ}t=0.01\tau_{\text{LJ}}$, and “snapshots” of the conformation are taken every $2\times 10^{3}\tau_{\text{LJ}}$; the first $10\times 10^{4}\tau_{\text{LJ}}$ are not used (to allow the system to reach a steady state). Such a set of 10 simulations typically takes around 4 days to complete using 10 compute cores running in parallel. For the hierarchical clustering analysis presented in Figures 4 and S9, 200 shorter simulations $\left(20\times 10^{4}\tau_{LJ}\right)$ were performed with only the final configuration being used.

We note that simulated Capture-C results depend rather weakly on the TF and LE parameters, but the simulated FISH results are more sensitive. Previous work on the loop extrusion model (Fudenberg et al., 2016) also showed that there is a complicated relationship between loop extruder parameters and the resulting simulated Hi-C maps. For all parameters we have chosen, where possible, values which are reasonable based on the literature, and give good predictions of the data; an exception is the LE parameters in the heteromorphic polymer model, and the rationale for doing so is discussed below. Due to the significant time it takes simulations to run we have not performed a systematic sweep of parameters; we note that the aim of the modeling is to understand the mechanisms behind organization of the locus, rather than to find a set of parameters which give results which exactly match the experimental observations.

For the simulations with the heteromorphic polymer presented in Figure 4, we found the length unit to be σ=21.8 nm, and the time unit to be approximately 0.5 ms. With this mapping the simulation run time was 250 s, the TFs switch at a rate $k_{\text{sw}}=0.25\;{\text{s}}^{-1}$ (or every 4 s), and the LEs move at $k_{\text{ex}}=4\;\text{kbp}\;{\text{s}}^{-1}$ and unbind with rate $k_{\text{off}}=0.05\;{\text{s}}^{-1}$ (or every 20 s). The latter LE parameters were chosen for reasons of computational efficiency and are not individually realistic (as unbinding and motion are too fast). However, the key control parameters in the model to determine most steady-state properties are the CTCF locations and LE processivity (Goloborodko et al., 2016) (the latter is given by the ratio $k_{\text{ex}}/k_{\text{off}}$ – here 80 kbp), and these parameters are all realistic.

##### Simulation Input Data

In order to simulate a specific region of the chromosome in different cell types, we used experimental data to determine the binding sites for the TFs. In Figures 2A and 2B we used the H3K27ac ChIP-on-chip data to infer TF binding sites: peaks were called from the data, and any bead representing a region which overlapped a peak was designated as TF binding. In Figure 2D and later plots, we instead used DNA accessibility data (ATAC-seq or DNase-seq) to determine the binding sites. Again, peaks were called from the data (Zhang et al., 2008), but here the bead which overlapped the center of each peak was designated as TF binding.

In order to identify beads as “loop anchor sites” we used CTCF and Rad21 ChIP-on-chip data. For each cell type we took the set of CTCF sites which overlapped with Rad21 peaks. Depending on the orientation of the CTCF binding motifs found within the peak (detailed above) we assigned the anchor bead as having forward, reverse or both orientations. In order to model cell-to-cell variation of CTCF binding we used the relative height of each peak to determine an occupation score – in each repeat simulation, each anchor bead was chosen to be present or not with a probability depending on this score. During LE dynamics, extrusion was halted when the direction of the anchor bead was opposite to the direction of motion – in this way persistent LE loops were only formed between convergent pairs of anchor beads.

##### Variable Compaction Chromatin Model

In Figure 4 we presented simulations from a polymer model which has a chromatin compaction (or fiber thickness) which varies along the polymer. As detailed in the main text and shown schematically in Figure 4A, this is achieved by adding additional springs between next-nearest neighboring beads along the chain, which has the effect of “crumpling” the chain into a thicker polymer. These i,i+2 springs were given by a harmonic potential

$$U_{\text{HARM}}\left(r_{i,i+2}\right)=K_{\text{HARM}}{\left(r_{i,i+2}-r_{0}\right)}^{2},$$

where $r_{i,i+2}$ is the separation of the next-nearest neighbor beads, $K_{\text{HARM}}$ is the bond energy, and $r_{0}$ the bond length. Since the i,i+2 beads now have springs connecting them, the extruders discussed above were initialised between i,i+3 beads. All other simulation details remained the same.

In order to obtain a fiber which varies in thickness along its length, we use the H3K27ac data to identify de-compacted regions, and do not include the i,i+2 springs in these regions. The persistence length of the de-compacted regions was determined by the Kratky-Porod interactions and set to 4σ as before. In the crumpled regions the Kratky-Porod interactions were still present in the model, but the harmonic springs dominate. We therefore cannot control the persistence length of the crumpled fiber in a simple way; we can estimate it by calculating the radius of gyration $R_{g}$ of a region of a uniform polymer of length L, plotting this as a function of L, and fitting the worm-like chain (WLC) formula. This is approximate since the polymer is self-avoiding and the additional springs may lead to kinks which give further deviation from the WLC. A further complication is that since we have a crumpled or zig-zagging chain of beads, we must find a smooth contour from which to calculate $R_{g}\left(L\right)$; since each bead is connected to four others (i−2, i−1, i+1 and i+2) we take the average position of groups of five consecutive beads which gives a smooth chain with measurable length. From this procedure we measured a persistence length of $l_{p}=4.7\sigma$ (the local thickness is also larger, and can be estimated as ∼1.75σ).

##### Simulated Capture-C profiles

In a Capture-C experiment, pairwise interactions between a given targeted *Dnp*II restriction enzyme fragment are sampled stochastically from a large population of cells. From our 13 targets and three cell types, on average each target had 107.2 normalized reads (reads per 100 million) from interactions within the chr2:105,000,000-106,000,000 region. To generate similar profiles from simulations we take a set of “snapshots” of the locus conformation (taken at regular intervals of $2\times 10^{3}\tau_{\text{LJ}}$ from at least 10 repeat simulations), and sample those stochastically. For each target bead we followed the following procedure: (i) select a chromatin bead at random; (ii) accept or reject this bead as interacting with the target with probability f(d), where d is the separation of the selected bead and the target; (iii) repeat this “interaction attempt” N−1 times (where N is the total number of chromatin beads). We used the following function for probabilities:

$$f\left(d\right)=e^{-d^{2}/d_{0}^{2}},$$

where we choose $d_{0}=3.5\sigma$ as the typical interaction length threshold.

This procedure is performed on each snapshot; the whole scheme can be repeated multiple times in order to obtain more simulated interaction “reads.” Importantly the same number of attempts are made for each target in each cell type, so the total number of accepted interactions for a given target reflects that target bead’s local neighborhood. Although in a Capture-C experiment we can only count accepted interactions, and normalize reads such that the total number of interactions is the same for each target, this normalization is done genome-wide. Thus, in the simulation scheme the fraction of attempts where an interaction is rejected represents interactions with loci outside of the simulation region.

To plot simulated Capture-C profiles alongside experimental data we scaled the simulated interaction count such that the total number of interactions within the *Pax6* region (chr2:105,000,000-106,000,000) over all targets in all cell types is the same in simulations and experiments. Importantly all profiles are scaled in the same way to preserve target-to-target and cell type variation.

##### Simulated FISH Measurements

Each FISH probe covers a region of the genome as indicated in Figure S3D and the table above; to generate probe separation measurements from our simulation data we identify which chromatin beads belong to each FISH probe and take the positions of the probes to be the center of mass of these beads. By taking regular $\left(2\times 10^{3}\tau_{\text{LJ}}\right)$ snapshots from each simulation we have a distribution of separations, in simulation length units σ, for a given cell type.

In order to map simulation length units to physical ones, we take six probe pair distributions from each of the three cell types (18 distributions in total). We use the two-sample Kolmogorov-Smirnov statistic to find the “distance” between each simulated distribution and the corresponding experimental distribution; we then sum these distances. We then numerically find the value of σ (to the nearest 0.01 nm) which minimizes the summed distances. Once the length scale has been identified, this allows determination of the simulation time unit as detailed above. For example, for the simulations presented in Figure 4 we find σ=21.8 nm and $\tau_{\text{LJ}}=0.5$ ms; similar values are obtained for the other simulation schemes. Since we were most interested in relative trends over cell types, in most figures we showed distances in simulation units.

##### Hierarchical Clustering

We previously showed that for the α and β globin loci a population of simulated conformations could be arranged into a small number of groups which have a similar set of chromatin interactions. To see if a similar grouping is observed in the *Pax6* locus, we performed a hierarchical clustering analysis. To do this we took a set of snapshots of the simulated locus conformations (taken after a run of $20\times 10^{4}\tau_{\text{LJ}}$ in 200 simulations) and calculated a “distance metric” for all pairs of conformations; there are several ways to calculate this metric (for example one could either consider just chromatin interactions, or one could consider the full polymer configuration). To perform the clustering one must also choose one of several “linkage” criteria, to determine how distances between groups of conformations are calculated. Hierarchical clustering can always build a dendrogram, but to interpret this one must ask how similar the dendrogram distances are to those of the underlying data, and whether any groups can be determined from this (and whether those have any physical meaning). Our strategy was to cluster using various combinations of distance metric and linkage criterion, and examine the results produced by each. We found that an *average* linkage criterion produced dendrograms with distances most similar to the underlying data (evaluated using the cophenetic correlation coefficient), and a distance metric based on the full polymer conformation gave informative clusters of conformations (i.e., they showed clear similarity within clusters and differences between clusters). The metric was defined as

$$\Gamma {\left(C,C^{'}\right)}^{2}=\frac{1}{n\left(n-1\right)}\sum_{i\neq j}{\left(d_{ij}^{\left(C\right)}-d_{ij}^{\left(C^{'}\right)}\right)}^{2},$$

where $d_{ij}^{\left(C\right)}$ is the separation of beads i and j in conformation C, and the sum runs over all pairs of beads. We perform the analysis considering an n=1000 bead region corresponding to chr2:105,000,000-106,000,000.

In Figure 5A we show some groups obtained from a hierarchical clustering of Pax6-HIGH cells: a majority (∼55%) of conformations fell into group E, in which the region forms a single “globule”; other groups showed conformations where the region formed two smaller globules (e.g., groups A,B,H), or a single globule but with some regions “looping out” (groups F,G,I). In general, the analysis of *Pax6* showed less clear grouping of conformations than was observed in our previous work on globin loci. That is to say, while groups were observed (Figure 5A), many of these only contained a few conformations, and there were many conformations which did not belong to any group. This suggests that the *Pax6* locus shows more variation than the globin loci. Particularly, going from the Pax6-HIGH, to the Pax6-ON and then to the Pax6-OFF cell types, fewer and fewer conformations could be placed into groups, suggesting that the configuration of the locus becomes less constrained as *Pax6* activity is reduced. Also, it is interesting to note that if we examined the interactions between the *Pax6* promoters and the distal regulatory regions (the URR and DRR), there was not much correlation with the overall configuration of the locus (Figure 5D).

It was also possible to directly group the populations of simulated conformations according to the interactions between the *Pax6* promoters and the URR and DRR (Figure 5E; if the separation was less than 6σ this was defined as an interaction). This gave five possible groups (a group where none of the three regions interact, three groups where two regions interact an a third does not, and a group where all three regions interact). As detailed in the main text we found that, consistent with the clustering analysis, the *Pax6* interactions did not depend on the size or shape of the locus (Figure 5F).

##### Transcriptional activity score

In Figure 5I we defined a transcriptional activity score from the simulated conformations based on the proximity of promoters and enhancers. Specifically, this was defined as

$$T=\frac{1}{d_{\text{URR}}}+\frac{1}{d_{\text{DRR}}}$$

where $d_{\text{URR}}$ and $d_{\text{DRR}}$ are the distances from the *Pax6* promoters and the URR and DRR respectively, and we assume that the promoter-enhancer interactions work additively (Hay et al., 2016). We then examine the distribution of log[T/〈T〉], were angle brackets denote mean over conformations, taking the standard deviation of this as a measure of transcriptional heterogeneity within the population. Importantly by normalizing by 〈T〉 we have scaled out the overall transcription level – we do not expect our simulations to be able to predict this level, since it will depend on the promoter/enhancer architecture (and our experimental results examining the DRR in Pax6-HIGH cells call into question the assumption that in this case enhancer action is mediated through physical contact between the enhancer and the promoter).

### Quantification and Statistical Analysis

In order to quantitatively compare the simulation results with experimental data we defined two measures, the Q-score and K-score which compare Capture-C and FISH results, respectively.

#### Q-Score

In order to quantify how well a set of simulated Capture-C interaction profiles agreed with experimental data we used a quality metric called the Q-score (Brackley et al., 2016a). We take the set of scaled interaction profiles generated as detailed above, truncate values lower than 0.35 to zero, and use a sliding averaging window to smooth both the simulation and experimental data, before applying a peak finding algorithm to identify interactions (the “findpeaks” function in the MATLAB software). We use the peak positions and widths (but not heights) to test whether peaks in each dataset overlap, and calculate a value

$$q_{ij}=\frac{n_{\text{se}}+n_{\text{es}}}{n_{\text{s}}+n_{\text{e}}}$$

for the i th viewpoint in cell type j, where $n_{\text{s}}$ and $n_{\text{e}}$ are the number of peaks found in the simulation and experimental data respectively, $n_{\text{se}}$ is the number of peaks in the simulation data which overlap with one or more peaks in the experimental data, and $n_{\text{es}}$ is the number of peaks in the experimental data which overlap with one or more peaks in the simulation data. It is possible for $n_{\text{se}}$ and $n_{\text{es}}$ to differ if, for example, two adjacent peaks in the simulation overlap a single broader peak in the experiment. To find the overall Q-score the average overall viewpoints and cell types were taken; the standard deviation of all the $q_{ij}$ values (when comparing a given simulation model to the experiments) gave a measure of the variation of agreement across viewpoints and cell types. From this definition we note that the Q-score can loosely be interpreted as the mean fraction of correctly predicted peaks in the simulated Capture-C profiles. In order to understand what different values of the score mean, we generated a randomized control dataset. This was done by assuming that the same number of Capture-C peaks as generated by the HiP-HoP model were randomly scattered within the locus (a peak at the viewpoint was assumed to always be present, and experimental and simulations peak widths were set equal to the average found in each experimental profile). By averaging the Q-score generated by many realizations of the random peak selection we obtain a value of 0.35 (compared to 0.51 for the HiP-HoP simulations shown in Figure 3C).

Although the Q-score provided a single number to describe the performance of a given simulation model, it is not a perfect measure – it is very difficult to quantify how well a given set of simulation profiles matches the data. For example, in the Q-score we use peak finding, but peaks in Capture-C profiles are not always well defined (there can be broad regions of interaction). Also, different models can show clear improvements in some respects (e.g., in Pax6-HIGH cells the model in Figure 2A shows broad interactions not present in the data (red stars), and these are absent in the model in Figure 2D), but perform less well in others (a number of new incorrectly predicted peaks appear in Figure 2D, so overall this results in very little change in the Q-score).

#### K-Score

In order to quantify the degree to which a set of simulations agrees with the FISH data, the 18 probe pair separation distributions were taken and the value of the simulation length unit which best fits the data was identified. The normalized two-sample Kolmogorov-Smirnov statistic describing the distance between each experimental and simulated distribution was then found. This takes a value between 0 (when the two distributions are identical) and 1 (when there is no overlap). The overall K-score is the average of these normalized distances subtracted from 1 (such that it takes a value 1 when the simulations are in complete agreement with the data). The K-score can therefore be interpreted as the average degree of overlap between the simulated and experimental probe pair separation distributions, The standard deviation of the individual Kolmogorov-Smirnov statistic values gives a measure of the variation of agreement across probe pairs and cells. In order to further elucidate the scale of K-score values, we generated a random simulated FISH dataset (probe separations were chosen from a uniform distribution with the same maximum and minimum values as found in the HiP-HoP simulations); this yielded a K-score of 0.59 (compared to 0.77 for the simulations shown in Figure 3C).

### Data and Software Availability

The accession numbers for the data reported in this paper are GEO: GSE119660, GSE119656, GSE119659, GSE119658, GSE120665, and GSE120666. The following data have been deposited in the Edinburgh DataShare (https://datashare.is.ed.ac.uk/): The processed simulation and experimental data used to generate all figures. The full set of 200 simulated locus configurations for each cell type used to generate plots and simulation snap-shot images for Figures 4 and 5, and Videos S1, S2, S3, S4, and S5. An input script and python driver script along with example initialization configurations which can be used to run a HiP-HoP simulation of the Pax6-HIGH cells using the LAMMPS software. Data analysis and simulation software used were all open source packages, as detailed in the Key Resources Table.

## Supplemental Information

Document S1. Figures S1–S6 and Table S1

Document S2. Article plus Supplemental Information
