# Supplementary material for: Polymer Simulations of Heteromorphic Chromatin Predict the 3D Folding of Complex Genomic Loci
Source: Mol Cell. 2018 Nov 15;72(4):786–797.e11. doi: 10.1016/j.molcel.2018.09.016 (PMC6242782; doi:10.1016/j.molcel.2018.09.016)
Supplement: Document S1. Figures S1–S6 and Table S1 [file mmc1.pdf]

Molecular Cell, Volume *72*

Supplemental Information

Polymer Simulations of Heteromorphic Chromatin Predict the 3D Folding of Complex Genomic Loci

Adam Buckle, Chris A. Brackley, Shelagh Boyle, Davide Marenduzzo, and Nick Gilbert

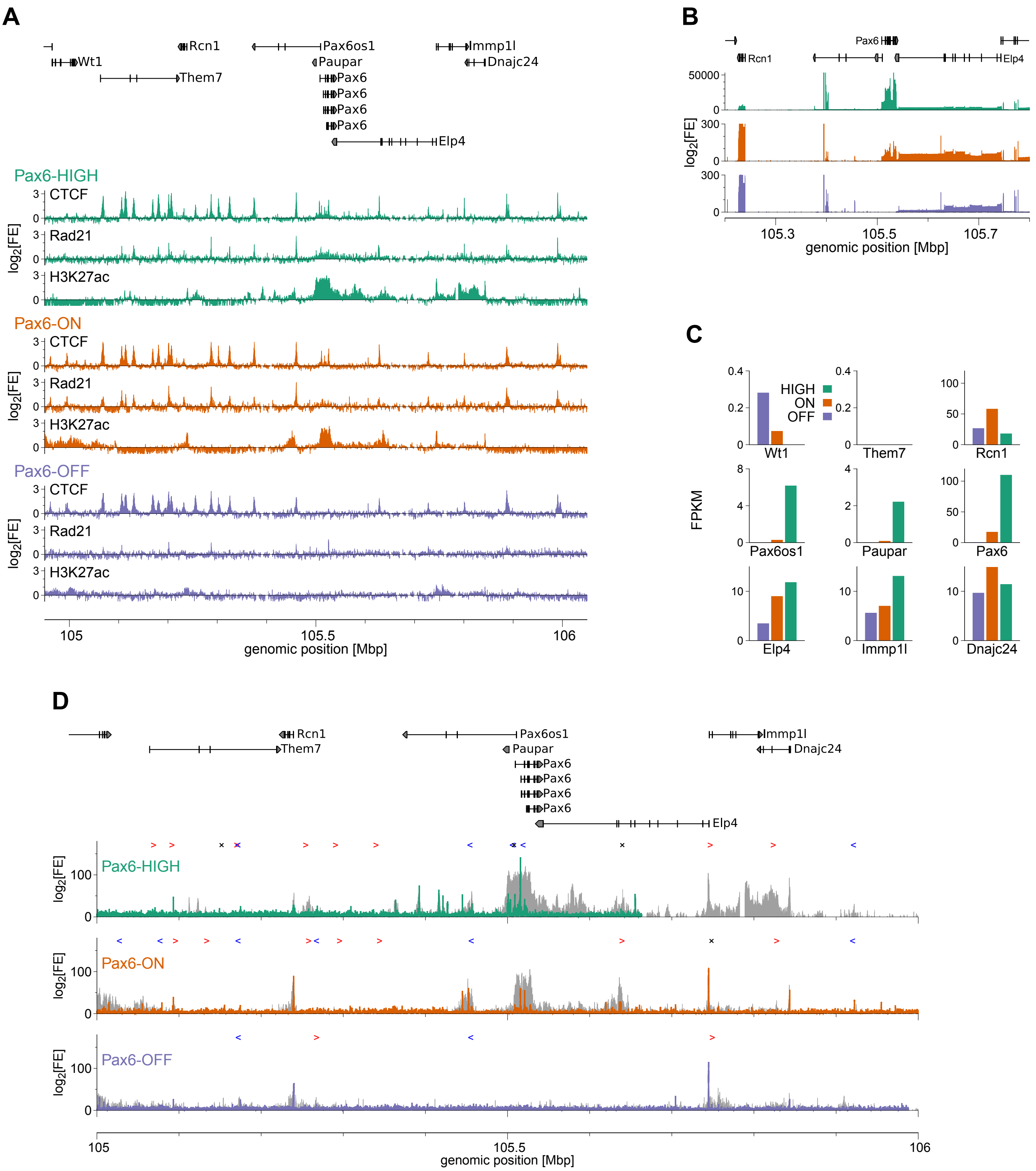

Figure S1. Epigenetic and protein marks across the Pax6 locus (related to Fig. 1).

(A) Top: Organisation of the Pax6 locus on mouse chromosome 2 (mm9 build). Bottom: ChIP-on-chip data for CTCF, Rad21 and H3K27ac in three Pax6 expressing cell lines (Pax6: OFF, ON, HIGH).

(B) RNA-seq signal in the vicinity of Pax6 for three cell types.

(C) RNA-seq data showing the expression level of each gene in the region for three cell types.

(D) Top: Genomic view of the locus. Bottom: Plots showing ATAC-seq data in three cell lines (coloured bars). Grey bars show ChIP-on-chip data for H3K27ac, and symbols above plots show positions and directions of CTCF peaks (which overlap with Rad21 peaks) identified from ChIP-on-chip data (red and blue arrow-heads indicate left and right orientated CTCF motifs, while black crosses indicates there are motifs on both strands; see Methods for details).

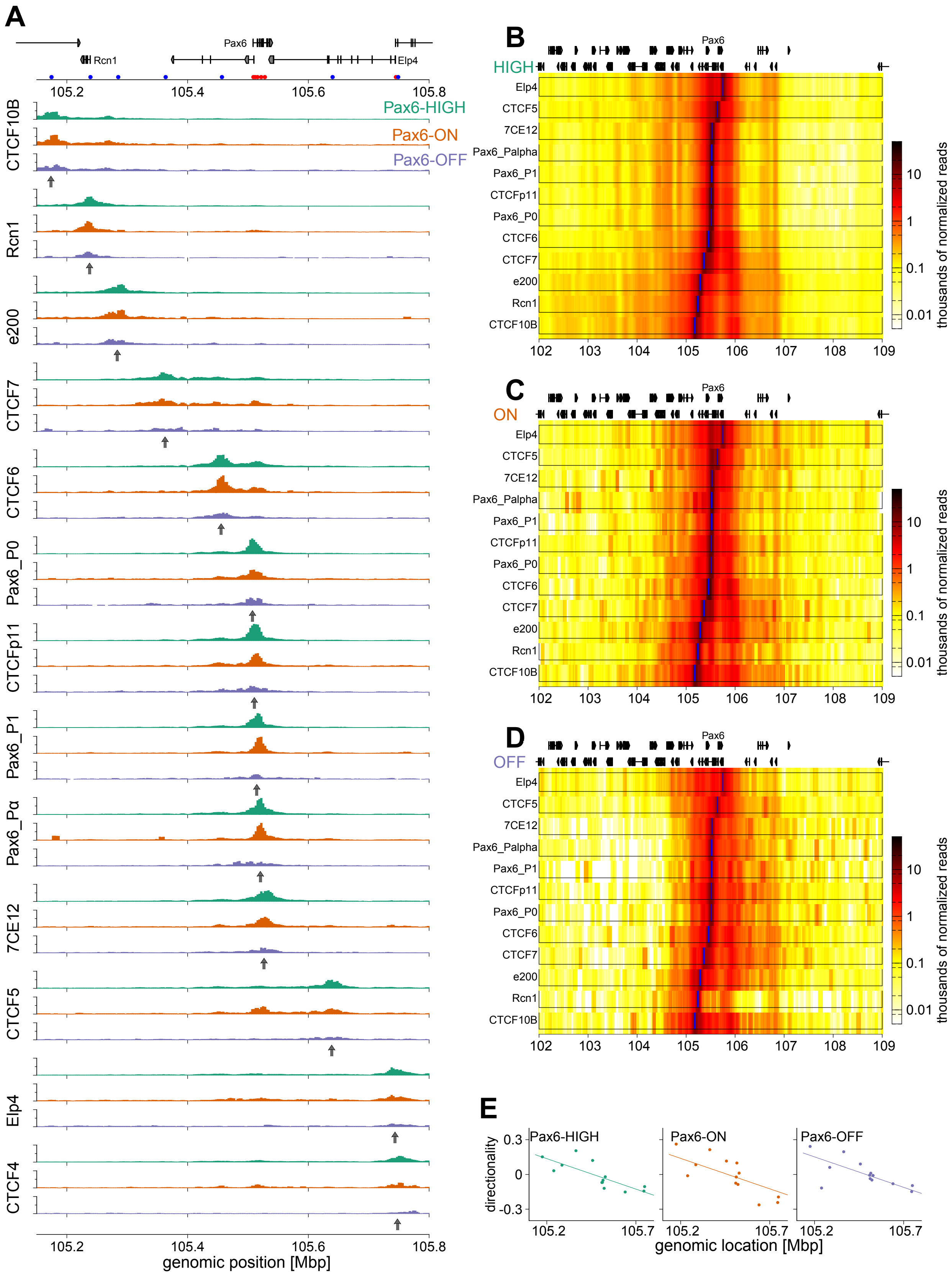

Buckle, Brackley, et al., Figure S2

Figure S2: Capture-C across the Pax6 locus (related to Fig. 2).

(A) Oligo probes were designed in two groups at 13 viewpoints across the Pax6 locus (red and blue points under browser view). Bar plots showing the Capture-C interaction profile for each oligo probe in each of the three cell lines. Vertical axis shows numbers of reads per hundred-million, and each plot shows the same range [0-7]. Data are smoothed using sliding window bins with window width 6 kbp and bin step 3 kbp. See Methods for details. Arrows under each set of plots indicate the location of the viewpoint.

(B-D) The same data are shown as heat maps for a larger region at a lower resolution (sliding window bins with window width 25 kbp and bin step 50 kbp). Viewpoints are shown as blue lines.

(E) Plots showing the directionality of interactions from each viewpoint, where the horizontal axis gives the position of the viewpoint. Directionality is defined as (downstream-upstream)/total interactions within a 2 Mbp window around the viewpoint. The solid line shows a linear fit to the data where the slope (which is roughly the same in each cell type) indicates that the locus sits in the middle of a domain.

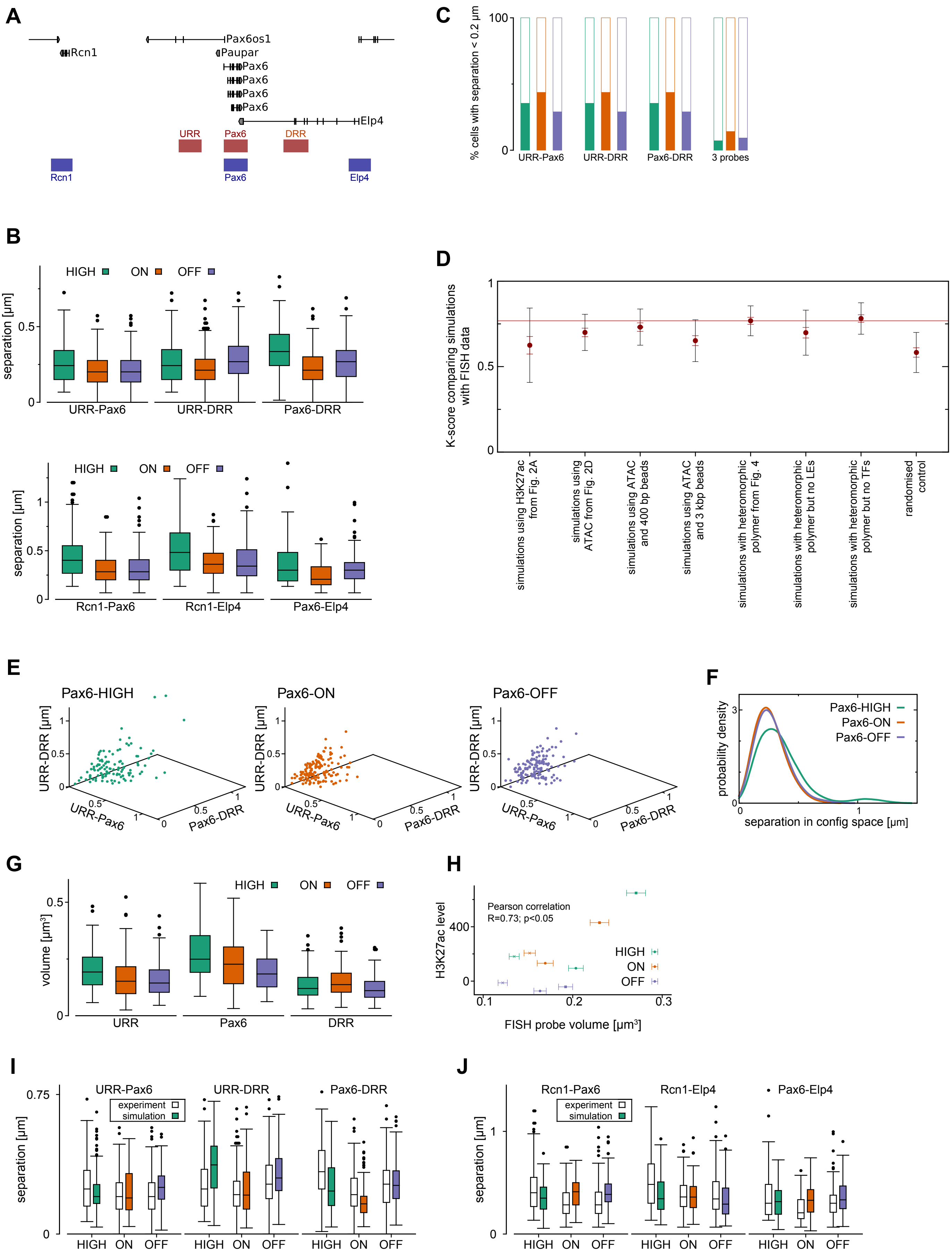

Buckle, Brackley, et al., Figure S3

Figure S3: Locus organisation at Pax6 characterised by FISH (related to Fig. 3).

(A) Map of the Pax6 locus (chr2:105,200,000-105,800,000; mm9 build) showing FISH probe locations. Three-colour FISH was performed for each of two groups of probes: probes covering the up and downstream regulatory regions along with Pax6 (red) and probes covering three promoters within the locus (purple).

(B) Box plots showing the separations of pairs of probes.

(C) A separation threshold of 0.2 μm is commonly taken to imply functional interactions. Here we plot the percentage of cells for which this criteria is met for each cell type. Figure 2C can then be used as a comparison between FISH and Capture-C results.

(D) Comparison of FISH measurements between different simulation models and experimental data. The K-score gives a comparison between a set of simulated FISH probe separation distributions and experimental data. This is based on the Kolmogorov-Smirnov statistic, and takes values between 0 and 1, where 1 indicates a complete overlap of all simulated and experimental distributions (see Methods). A K-score for each the of different sets of simulations of the Pax6 locus is shown. Points represent an average over scores for each distribution of probe pair separations in each cell type; the red error bars show the standard error in the mean. The black (larger) error bars show the standard deviation, giving an indication of the variation across probe pairs/cell types. The dotted line shows the K-score for the final simulation model which includes TFs, LEs and the heteromorphic polymer (the simulations presented in Fig. 3).

(E) Since there are simultaneous measurements of three probe separations in each cell, we can show each cell as a point on a 3-D scatter plot. The points in this 3-D "configuration space" can be used to calculate a locus size measure for each cell (distance of the point from the origin), and a measure of the cell-to-cell variability (the volume occupied by the cloud of points; see Methods).

(F) Plot showing the distribution of the separation in pairs of points from (E).

(G) Box plots showing the distributions of the volume of each probe.

(H) Plot showing the measured volume for each probe in each cell type against the level of H3K27ac within the probed region (mean log[FE]). Error bars show the standard error in the mean of the FISH probe volume distribution. The Pearson correlation coefficient is $R=0.73$ ($p<0.05$). Since the length covered by each probe is approximately the same we have not corrected for this. Colour indicates cell type while point symbols indicate probes (circles, squares, and crosses indicate URR, Pax6, and DRR probes respectively).

(I-J) Simulated FISH probe separation distributions are shown as box plots (filled boxes) side by side with experimental data (empty boxes). Here the simulation length unit was found to be 21.8 nm as detailed in Methods.

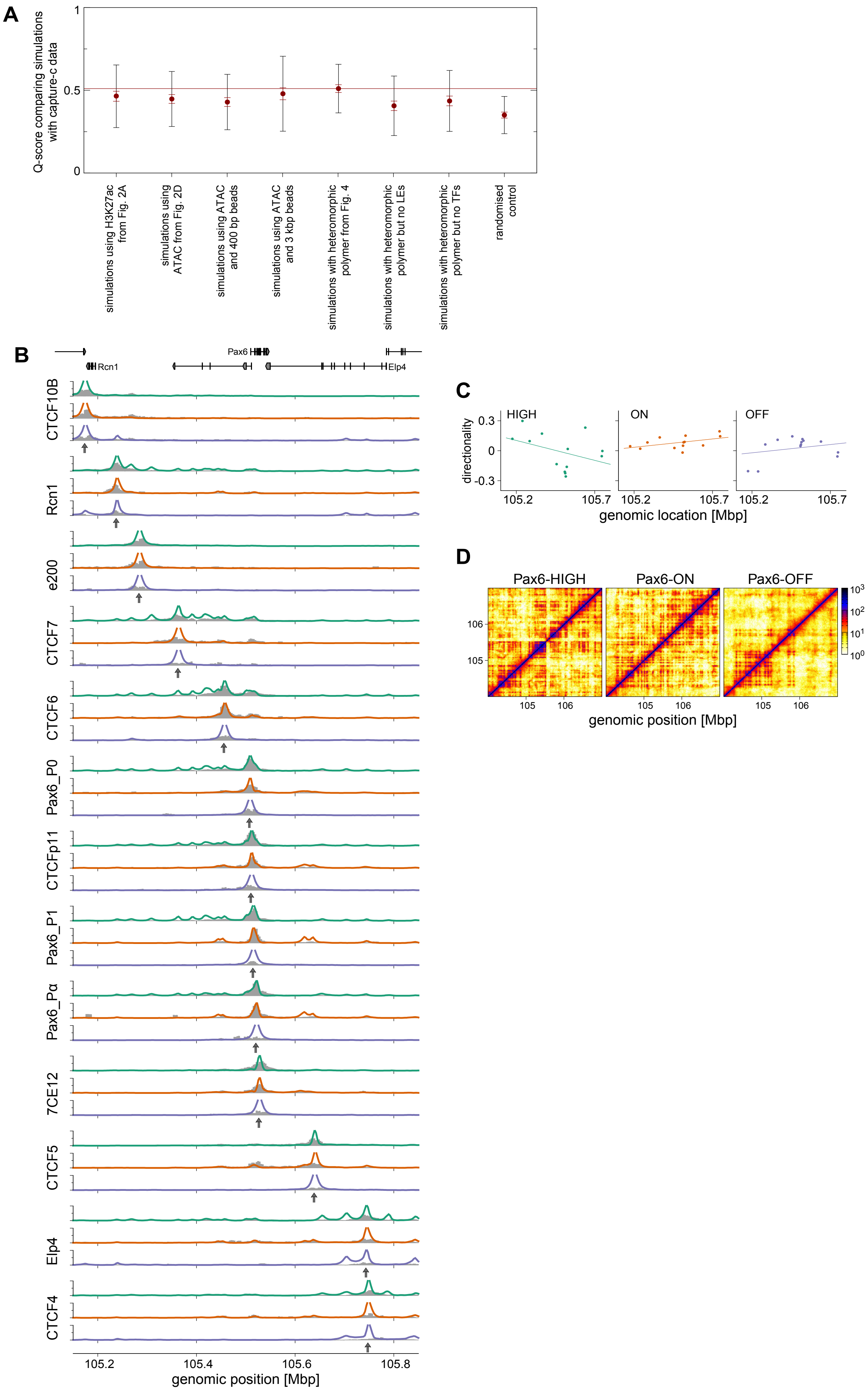

Buckle, Brackley, et al., Figure S4

Figure S4. Comparison of Capture-C interaction profiles between heteromorphic polymer simulations and experimental data (related to Figure 4).

(A) The Q-score gives a comparison between a set of simulated Capture-C profiles and the corresponding experimental data. This takes values between 0 and 1, where 1 indicates a complete overlap of simulated and experimental interaction peaks (see Methods for details). A Q-score for each of the different sets of simulations of the Pax6 locus is shown. Points represent an average over Q-scores for each Capture-C profile (viewpoint) in each cell type; the red error bar shows the standard error in the mean. Black (larger) error bars show the standard deviation, giving an indication of the variation across viewpoints/cell types. The dotted line shows the Q-score for the final simulation model which include TFs, LEs and the heteromorphic polymer (the simulations presented in Fig. 3).

(B) Plots showing simulation vs experimental Capture-C profiles for all oligo probes. Solid lines show simulated Capture-C profiles with grey bars showing experimental data. Viewpoints are indicated with arrows.

(C) The interaction directionality measure presented for experimental data in Fig. S2e can also be extracted from simulations. Points show the directionality for each viewpoint, and the solid line is a linear fit.

(D) Simulated HiC maps across the wider Pax6 region in each cell type.

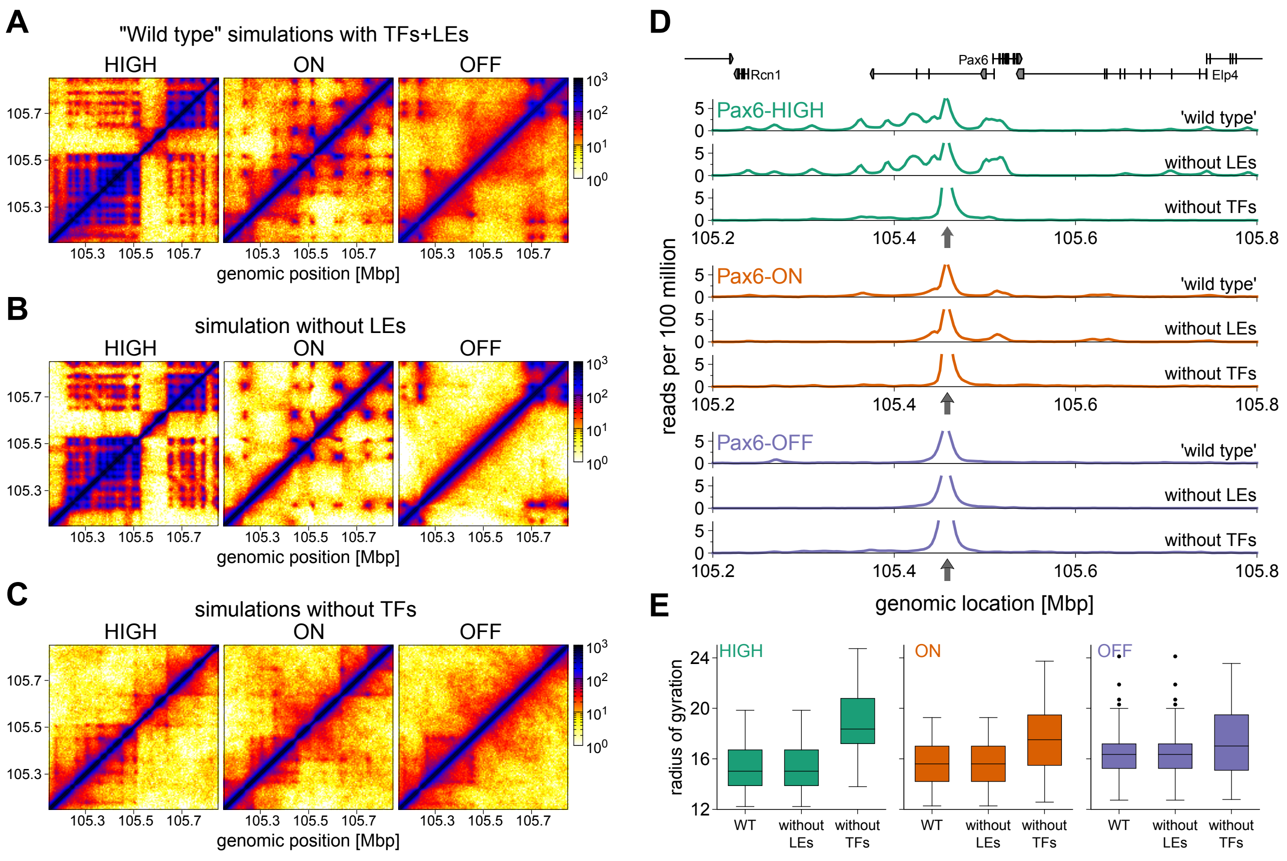

Figure S5: Model variations (related to Figure 4). To test different scenarios, parts of the model can be switched off; here we examine the case of no LEs (equivalent to a cohesin knockout) and the case of no TFs.

(A) Simulated HiC maps showing interactions within the Pax6 region for each cell line for the "wild type" case (all model aspects present).

(B) Similar plot to A but LEs were turned off.

(C) Similar plot to A but here TFs were switched off.

(D) Representative simulated Capture-C profiles are shown for one view point (CTCF6) for the three cases in each cell line.

(E) Box plots showing the distribution of the radius of gyration of the region chr2:105,000,000-106,000,000 for each cell line.

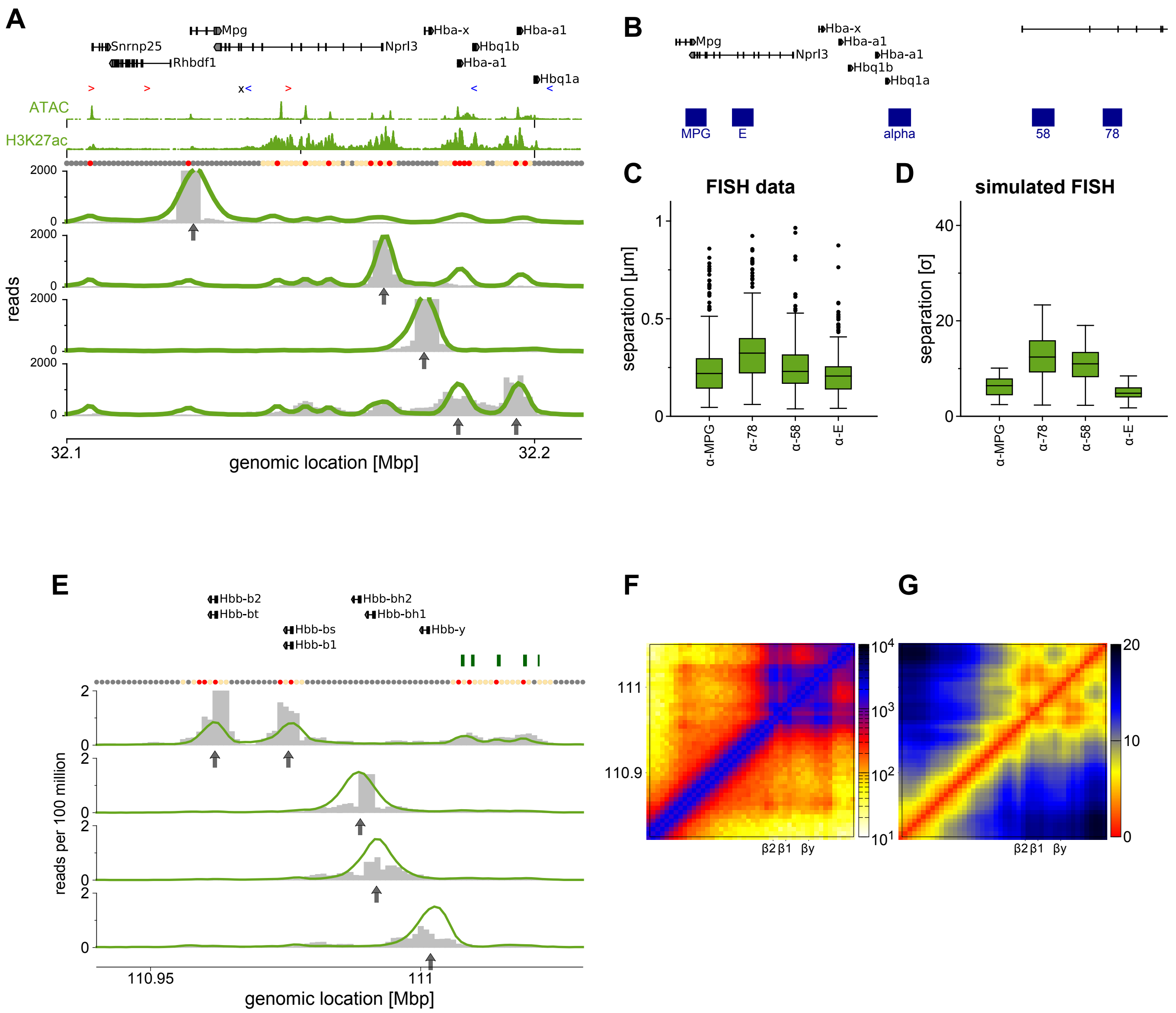

Buckle, Brackley, et al., Figure S6

Figure S6: The α and β globin locus. Results from simulations of a 2~Mbp region around the α and β globin genes in mouse erythroid cells using the heteromorphic polymer model (related to Figure 6).

(A) Top, the α-globin locus and the data used as simulation input, and points indicating the bead colours. Capture-C data from four viewpoints (indicated with arrows). Simulation data is shown as a solid line, whereas experimental data (from Hughes et al., 2014) is shown as grey bars. Since there are two copies of the α globin gene, oligo captured fragments could have come from either copy, hence two viewpoints are shown on the same plot.

(B) Map of the locus showing positions of the FISH probes.

(C) Experimental FISH data (Brackley et al., 2016a).

(D) Simulated FISH data using the same probe pairs.

(E) Top, the β-globin locus and below Capture-C data from four viewpoints (indicated with arrows). Simulations are shown with solid lines, whereas experimental data (from Hughes et al., 2014) are shown as grey bars. Since there are two copies of the β globin gene, oligo captured fragments could have come from either copy, hence two viewpoints are shown on the same plot. Above the plots green boxes indicate the locations of known enhancer elements. The row of points indicates bead colourings: red are ATAC-seq points, yellow indicates H3K27ac regions.

(F) Simulated HiC map indicating β -globin interactions.

(G) Map showing average separation from simulation data between fragments (simulation length units, σ).

Table S1: Pax6 Capture Oligos. Oligos were 5' biotinylated and a pair of oligos was used for each genomic site (related to STAR method).

Pax6_P1 (a)	GATCAAGTTCGCGGCCGCAGCGCTGGCCGCCGAAGCGCATGGAGCGGGAGACCTCGGCGAGCG CCAGAGCCTAGGAGCGGGGGGCCCAGCGCCGGAGAGAGAAGCCGGGACCCACCGGCA
Pax6_P1 (b)	CTTGAGCCATCACCAATCAGCATAGGTGTGCTGGCTGCAGCCACTTCCCCACACTCTTTATCTCTCA CTCTCCAGCCGCTGACAGCCCATTTTATTGTCAATCTCTGTCTTCTTCCCAGG
Pax6_P0 (a)	GATCCGCGTACTGGATGGCCCCTGAACTCCGCAGGACCTGTTTACTTGAAAGTAGGGGGAGGGG GGCTTAAGCCGAACCTCAGGGAGGACAATACCAGCCAGAGGCAGGCTGGGCGTGTG
Pax6_P0 (b)	ACCAATGAGGGCATTGCTGGCGTGGATATTAAGGAAAGTTAGCGCCTGCCGGAGCACCCTCTTTT CTTATCGTTGACATTTAAACTCTGGGGCAGGTCCTCGCGTAGAACCCGGTTGTCA
Pax6_Palpha (a)	GATCCGTACCCTGGAGCTTGGACTCCTTAGCCTTAGGTTTTCAGGTGGGCCGAGGCGCTCAGTCG CGACCGTTTGCCGCTACACTATGGCCTGGCTGGCAGGCCCAGAGGGGTACGGGTC
Pax6_Palpha (b)	TGGGAATTACCCTGGCTTTGCTTTTGAAAAGTTTCTCTTCATTTCTCTGGGAAGGCCTTTCTTTTTC TGGGAAGCTTTCTTTTGCCCAGAGATGAGCCTCCATACCTGCATGGTAACTGA
CTCFp11 (a)	GATCCCTTGCTACTGCTGCCTCTGCTCTCCAGTCACTAATAAAACTCGCATTGAGCCCCTTAGTGCC TTGATTAACAGGCAGATTAACTCTTACTGGGGTTGGGAACAGCTTGTCTCAAT
CTCFp11 (b)	AGGCAGGTTGCTGGGGTAAGCGGAGAGAGAAAGCCCCAAGCCAGGGAGGCATCAGGAACTGG GTGGCTAGGGTGGAAGACTTTGATTTTTATGACCTTTTGATTTGTTTGCCATCCCAGA
7CE1/2_CTCF (a)	GATCTAGGAGGGCTGAGTTCAGAAGCCCTCCACTCCTGTGCTCCCTGGCGTTTGGGGCTCAGGTT TGCATGGAGACCAGGGAATGAACACCCTCTTCCTGTCTCTGGGTGCCAAGACTAA
7CE1/2_CTCF (b)	ATTTTCCCCCAGGCTCTCTGCTCCGCCTCCAGTGTGGGGAGGAAGAGTGGCTGGAAAGGAGAAT CTTGGGCGTGGGGACTTCGGTCTGTGGTGTCAGCTATCCAGGCTTAAATTCCTGGG
Elp4_Pro (a)	GATCCTACAAGCCAATTCCTTTCCATATGTGTTTCTTTTTCAGTAGTAATCATTTTAGGGTAATGGCA TGTAGGGAGTCCATGGTCATCTAGAAATGAGGTGCTGAACAGAGAAGAGTAA
Elp4_Pro (b)	GTTAAGTGATTTCTAGAAGACATCCAAGATAAGCAATGTTTGAACGGAAAAAGAGACCTAAGTCA ATACTTGTGTCACTTAATTACTGCTGACAGTGATGGGTGATACATGATATATGGA
CTCF5 (a)	GATCAACTGTGTACTCTAACTTTGTTTTGAAAAGGATGGTTCTTCATTTGTTTTAGAGCATGATTTT AGAAATACCATTTGTTTAAACTGACAGAGTAGATATTTATGAGTTTTGCCTTT
CTCF5 (b)	TTTTCTTTCATTATTTAAGATATAACCAATAAATTATTTAAGGTGTCCCCTTTCAGTCTTCTGTATCCA CACGTCTTCTAATACCCTACTTTTAGCATACGAATTCTTCTAATATATAGG
CTCF6 (a)	GATCTGAAGCTGTGTAGACAGAAGCAGGCGGGCCACAGTTACAGGCCTTACTGTATCATTTACC AGCTGGTCACTCGGACCATCCATGTCTCTACTGCACAGTGACAATTAGCATCCCTT
CTCF6 (b)	ATAGTACCTGGCTCTGTTTCCTGGAGTGGAGAAGGGCCTGCACAAAAACTCATTGAATCCAAATA AAGTCTAGCTTAAACAATACTATTACCAATGTCAATTTGTTAGGGGTTTTGATAG
CTCF4 (a)	GTGAGCAAAGAACTGCTCTGTGCCCCACCCCCTTGGTGGGGGCGGGGACATTGTTATAGTCACA AAAGTGCTAGACTGTTGCCACCAGGTTAATAATCTACTGAAGTCTTATGGGAGGAC
CTCF4 (b)	CATAAAGTTGAACAAATTGTAGAACAGACTTATGGTAAGTAGAATTTTTAGAATTCAAACACCCCT TGATTTCCTTTATGGGTTTCGTATTCAAGTTAAGTCCTTGGCTGTTGTAAGGCT
CTCF7 (a)	GATCATACATTGTACCGCTTCATCACAATATGCTGCTGGAGTATCAGTATTGTTCATATTTATAGAAG AGGCGAGCAGCCCAAAGAGGTTAAGTAACTTGCTTAAACCTTGACAGCGACT
CTCF7 (b)	TTGGATGAACTCCCAGAGGTCACATCCATCTGGGGCTTGGTGGTTTTGCCTGATGCTCAACCAGT GTTGGCGTGGCCTCTTTCTCAGAGCCTAGCAGGAGACTCATAGCCGGCATTTTCA
CTCF10B (a)	GATCTTAAAGCTTCCCACCTTTCATGGAGCTCTGACGTAAGAAGCCCTTTCTGAAGACCTCTGTA CCTTTCACTAGACTGTCATTAGATGTGGTGACAGTCCTCCTACTCGGTGGCCTTT
CTCF10B (b)	AGGATTATTAGAGGCTTCAAAGAATTCTGGTTAATGAATCTTTGACTTGCTTTTCTTAGGTTTGTTT AAAAACAATCATACTTTTATCCAGTCTTAAGTTTGTGTCTCTTTCTCTTTCCC
Rcn1_pro (a)	GATCTTGAGATGTTTTTAAGCAAGTCTCAAGTGCCTCTCTCTGGACTATGGGAAAGAACCAGGTA CTAAAAGGCCACAGCCAACAAACTCTGGCTCACTCCAGTCACTTCTGCTGAAACC
Rcn1_pro (b)	CTTTGCGCTTGTGGTCCCGCGTGGCTCCCTCTGAGGTGGCCATGGGCCCCTGCGTGGTCCCCGG GCGGCCCGGGCCGCAGCCTCACCCCAGCCTCTCCTTGCTCTCGTCCGGGCTTAGCT
E200_enh (a)	GATCCACAATTTGTTCTTTCTCTAAACAGAGTCAGTTTTAGATAACCAGGTTGGCTCTTCTAAGA ATGCGACCAAGAAACAGAAGCTGGTAGTGAATGGTGGAATTGAGAATCCAAGACA
E200_enh (b)	GAACATGGTTAGCCTCAGCGTTTCTGGTCCAGCACATTTGAAGCAGGGCTGTAGTTGGATTGTTG ATGTGTTTACTGGAGACTCTGAATGTTTACACCAAACTCCACTCATACTATCATT
